# PST‐24: A Zeolite with Varying Intracrystalline Channel Dimensionality

**DOI:** 10.1002/anie.202007804

**Published:** 2020-08-11

**Authors:** Donghui Jo, Jingjing Zhao, Jung Cho, Jeong Hwan Lee, Yang Liu, Chang‐jun Liu, Xiaodong Zou, Suk Bong Hong

**Affiliations:** ^1^ Center for Ordered Nanoporous Materials Synthesis Division of Environmental Science and Engineering POSTECH Pohang 37673 Korea; ^2^ Berzelii Center EXSELENT on Porous Materials Department of Materials and Environmental Chemistry Stockholm University 106 91 Stockholm Sweden; ^3^ Co-Innovation Center of Chemical Science & Engineering School of Chemical Engineering and Technology Tianjin University Tianjin 300072 China

**Keywords:** aluminosilicates, channel dimensionality, electron diffraction, structure elucidation, zeolites

## Abstract

Herein we report the synthesis, structure solution, and catalytic properties of PST‐24, a novel channel‐based medium‐pore zeolite. This zeolite was synthesized via the excess fluoride approach. Electron diffraction shows that its structure is built by composite cas‐zigzag (cas‐zz) building chains, which are connected by double 5‐ring (d5r) columns. While the cas‐zz building chains are ordered in the PST‐24 framework, the d5r columns adopt one of two possible arrangements; the two adjacent d5r columns are either at the same height or at different heights, denoted arrangements S and D, which can be regarded as open and closed valves that connect the channels, respectively. A framework with arrangement D only has a 2D 10‐ring channel system, whereas that with arrangement S only contains 3D channels. In actual PST‐24 crystals, the open and closed valves are almost randomly dispersed to yield a zeolite framework where the channel dimensionality varies locally from 2D to 3D.

## Graphical abstract

PST‐24, a medium‐pore zeolite with a disordered arrangement of double 5‐ring (*d5r*) units, has been synthesized under excess fluoride conditions. Its three ordered polytypes have different channel dimensionalities, depending on the *d5r* arrangement order.

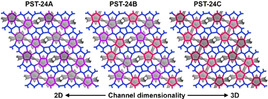

## Introduction

Zeolites and related microporous materials are among the most important classes of industrial catalysts and adsorbents.^1^ To discover new zeolite structures with unprecedented shape and/or surface selectivity properties, many rational synthetic strategies, like the long‐lasting organic structure‐directing agent (OSDA) design approach, have been developed.^2^ Despite the fact that a huge number of chemically feasible hypothetical zeolite structures have already been proposed, only 252 framework types are included in the Database of Zeolite Structures by the Structure Commission of the International Zeolite Association.^3^ This implies that there is still a strong need to develop more effective approaches for synthesizing unprecedented structures, especially materials with the aluminosilicate composition that show high Brönsted acidity and structural stability, which are of industrial relevance for catalysis and adsorption.

Since the seminal work of Flanigen and Patton in 1978,^4^ the use of fluoride anions first as a mineralizing agent and later on as an inorganic structure‐directing agent has been of major interest in zeolite synthesis.^5^ This is particularly true when the structure‐directing effect of Ge toward the formation of double 4‐ring (*d4r* or [4^6^]) units is properly combined with that of an OSDA in concentrated fluoride media (H_2_O/SiO_2_<10). Over the past two decades, in fact, a large number of new zeolite structures containing *d4r* units have been discovered.^5^ The molar concentrations of F^−^ and OSDA in the synthesis mixture are generally identical to each other, but F^−^ encapsulation within various types of small cages like *d4r* units is competitive with Al substitution in the zeolite framework. This led us to hypothesize that increasing the F^−^ concentration in aluminosilicate synthesis mixtures containing a particular OSDA can alter the phase selectivity of the crystallization. Indeed, we have recently been able to discover three novel aluminosilicate zeolites, i.e., PST‐21 (PWO), PST‐22 (PWW), and PST‐30 (PTY), using this so‐called excess fluoride approach.^6^

Most zeolites with disordered structures are characterized by one‐dimensional (1D) stacking disorder of the same building layers, leading to a partial peak broadening in their powder X‐ray diffraction (PXRD) patterns.^7^ Different stacking sequences of the same building layer can also result in different ordered zeolite polytypes. A common feature of zeolites generated by different layer stackings is that their channel dimensionality is preserved.^7^ On the other hand, the diffusion path length in zeolites, which has been mainly adjusted by the control of crystal size and mesoporosity so far, is a crucial factor governing their catalytic and adsorption behavior. One excellent example is that a large (3 μm) ZSM‐5 (MFI) crystal has significantly higher *p*‐xylene selectivity in toluene alkylation and disproportionation than a small (0.5 μm) one.^8^ Nevertheless, the control of intracrystalline diffusivity in zeolites by changing the channel dimensionality has never been reported. Here we present the synthesis, structure solution, and catalytic properties of a new medium‐pore zeolite with varying intracrystalline channel dimensionality, denoted PST‐24.

## Results and Discussion

**Figure 1:**
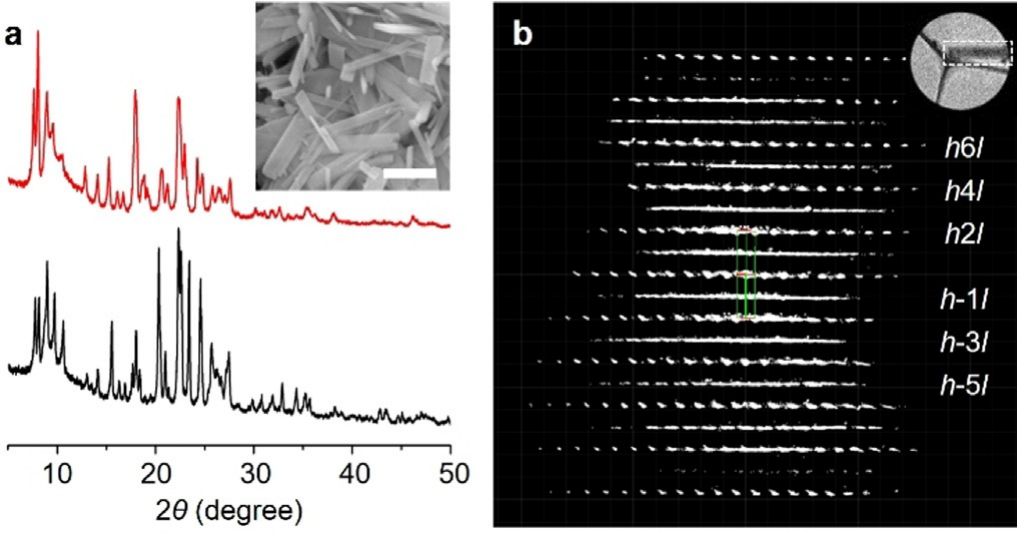
a) PXRD patterns of the as‐made (black) and calcined (red) forms of pure‐silica PST‐24. Inset: SEM image of its as‐made form (scale bar: 1 μm). b) 3D reciprocal lattice of calcined, pure‐silica PST‐24 reconstructed from cRED data viewed along the *c*‐axis. While the diffraction spots with *k*=2*n* are sharp, those with *k*=2*n*+1 are shown as diffuse streaks. Inset: the crystal where the cRED was collected.

PST‐24 was found when the pentamethylimidazolium (PMI^+^) cation is employed as an OSDA in highly excess F^−^ conditions. Table 1 lists the products from the PMI^+^‐mediated synthesis of zeolites using reaction mixtures with different fluoride compositions. It can be seen that when the HF/PMIOH ratio in the reaction mixture was fixed to 3.0, the Si*/*Al ratio leading to pure PST‐24 formation was 50 or higher (Figure 1 a). When the HF*/*PMIOH ratio was 2.0 or lower, however, SSZ‐50 (RTH) and HPM‐1 (STW) were the zeolite phases that crystallized at Si/Al=5–20 and ∞, respectively, as already reported.^9^ PST‐24 formation is observed at fairly lower Al and higher F^−^ concentrations compared with prior crystallization conditions (Si/Al=10 and HF/OSDA^*n*+^=2*n*, where OSDA^*n*+^ is 1,2,3‐trimethylimidazolium, 1,2,3,4‐tetramethylimidazolium, and 1,1′‐(1,4‐butanediyl)bis(2,5‐dimethyl‐1*H*‐pyrazol‐2‐ium) ions for PST‐21, PST‐22, and PST‐30, respectively).^6^ It thus appears that the compositional range in which the excess fluoride approach is viable for finding novel zeolite structures is rather wide. In particular, the fact that the use of a pure‐silica reaction mixture with HF/PMIOH=3.0 gave PST‐24 instead of HPM‐1 implies that the structure‐directing ability of OSDAs can differ significantly according to the concentration of F^−^ ions, even in the absence of heteroatoms like Al.

**Table 1:** Zeolite synthesis results using PMI^+^ as an OSDA at different HF concentrations and Si/Al ratios.^[a]^

			Product^[b]^		
Si/Al	HF/R=0.5	1.0	2.0	3.0	4.0
5	A	SSZ‐50	SSZ‐50	SSZ‐50+(U)	
7.5	SSZ‐50	SSZ‐50	SSZ‐50	A+SSZ‐50	
10	SSZ‐50	SSZ‐50	SSZ‐50	SSZ‐50+A	D
20	SSZ‐50	SSZ‐50	SSZ‐50+U	PST‐24+A	
50	SSZ‐50 +HPM‐1+A	SSZ‐50+U	SSZ‐50+U	PST‐24^[c]^	D
∞	HPM‐1+A	HPM‐1	HPM‐1 +(PST‐24)	PST‐24^[d]^

[a] The composition of the synthesis mixture is 0.5 ROH⋅*x* HF⋅1.0 SiO_2_⋅*y* Al_2_O_3_⋅5.0 H_2_O, where R is PMI^+^ and *x* and *y* are varied between 0.25≤*x*≤2.0 and 0≤*y*≤0.1, respectively. All syntheses were performed under rotation (60 rpm) at 175 °C for 14 days unless otherwise stated. [b] The product appearing first is the major phase, and the product obtained in a trace amount is given in parentheses. A, U, and D indicate amorphous, unknown, and dense phases, respectively. [c] The Si/Al ratio of product was determined to be 200 by elemental analysis. [d] Obtained after 7 days of heating.

The SEM image reveals that as‐made, pure‐silica PST‐24 (Si‐PST‐24) are typically rectangular plate‐shaped crystals with ca. 0.06×0.25×1.5 μm^3^ in size (Figure 1 a). The ^13^C NMR spectrum of as‐made Si‐PST‐24 shows that the occluded PMI^+^ ions remain intact (Supporting Information, Figure S1). The framework of PST‐24 maintains structural stability after calcination at 600 °C, which was verified by PXRD, thermal analysis, N_2_ adsorption, and ^29^Si MAS NMR (Figure 1 a; Supporting Information, Figures S2–S4). Notable differences between the ^29^Si NMR spectra of the as‐made and calcined forms of PST‐24 suggest that its local framework structure has been substantially altered upon removal of occluded OSDA molecules during calcination at 600 °C (Supporting Information, Figure S4).^10^ On the other hand, the PXRD pattern of calcined PST‐24 shows peak broadening in the 2*θ* range from 9° to 12° (Figure 1 a), indicating the presence of structural disorder. Therefore, 3D electron diffraction (3DED) and high‐resolution transmission electron microscopy (HRTEM) were applied for the determination of the PST‐24 structure (see the Supporting Information for more details).

The 3D reciprocal lattice of PST‐24, reconstructed from the continuous rotation electron diffraction (cRED)^11^ data from a calcined, pure‐silica PST‐24 crystal, is characterized by alternating planes of sharp spots and diffuse scattering (Figure 1 b; Supporting Information, Figure S5), revealing the existence of disorder. Therefore, we decided to determine its average structure by using the sharp spots only. The unit cell parameters of the average structure determined from the cRED data were *a*=24.140(44) Å, *b*=5.211(18) Å, *c*=21.761(30) Å, *α*=90°, *β*=111.389(180)°, *γ*=90°. Based on the 2D slices cut from the 3D reciprocal lattice, the reflection conditions were deduced to be *hkl*: *h*+*k*=2*n*, 0*kl*: *k*=2*n*, *h*0*l*: *h*=2*n*, *hk*0: *h*+*k*=2*n*, consistent with three space groups: *C*2 (no. 5), *Cm* (no. 8), and *C*2/*m* (no. 12). The average structure could be solved and refined from the cRED data using the space group *C*2/*m* and the program SHELX (Supporting Information, Table S1).^12^, ^13^ Rietveld refinement was also applied on the synchrotron PXRD data (Supporting Information, Figure S6 and Tables S2–S5), while excluding the broad peak region (2*θ*≤12°).^13^ The final refined unit cell parameters were determined as *a*=23.7125(6) Å, *b*=5.0848(2) Å, *c*=21.3024(6) Å, *β*=111.838(2)° for calcined PST‐24.

**Figure 2:**
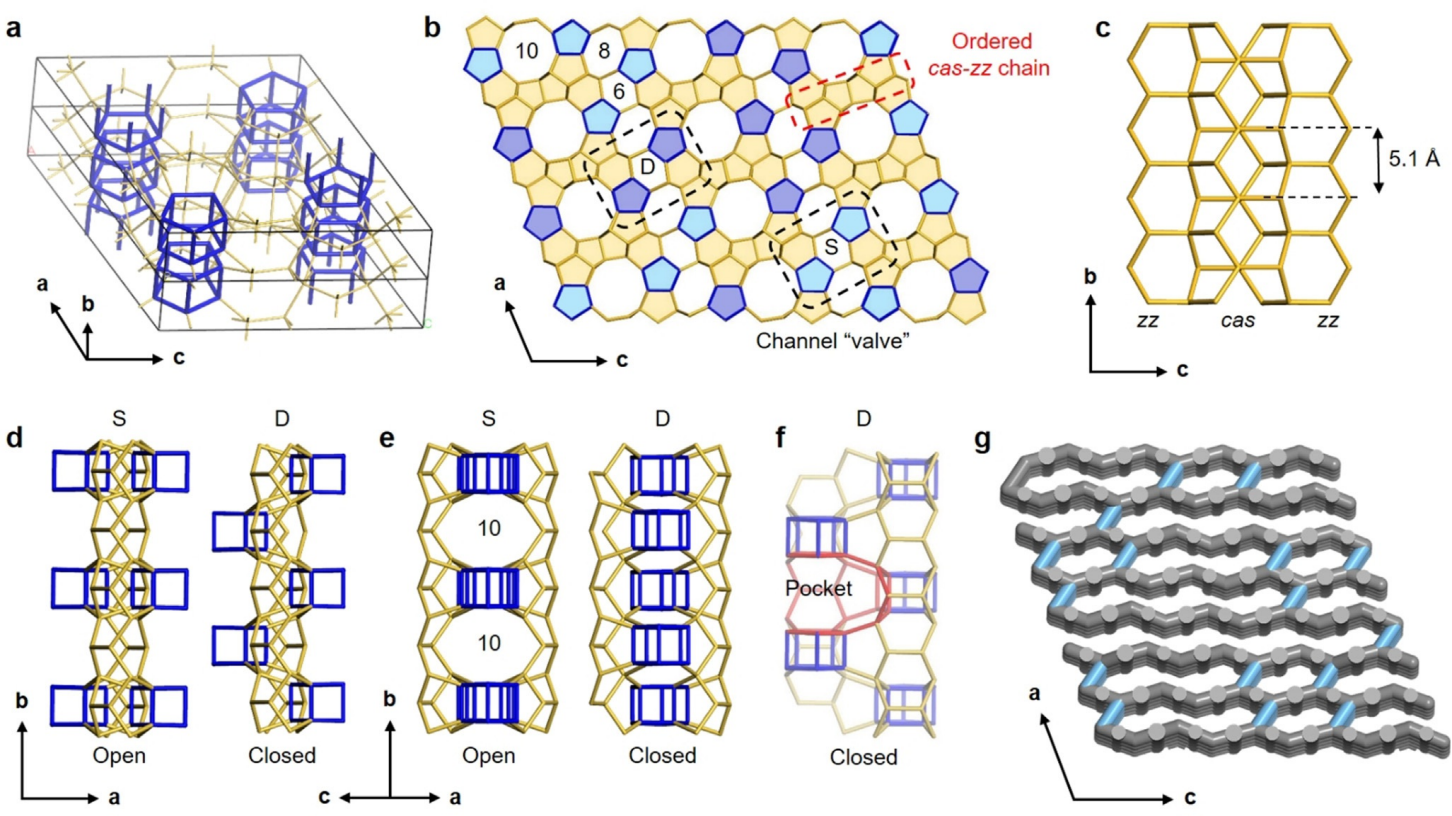
a) The average structure model of PST‐24 in 3D showing both the ordered *cas‐zz* chains (in yellow) and superimposed *d5r* columns (in blue). The *d5r* units in the same column are too close to each other so that only every second *d5r* unit is allowed in reality. Bridging O atoms have been omitted for clarity. b) One example of the real structures of PST‐24 projected along the *b*‐axis. The ordered *cas‐zz* chains are highlighted in yellow. The dark blue and light blue colors present the pairs of most adjacent d5r columns with the same (S) and different (D) heights, respectively. Arrangements S and D are randomly distributed in PST‐24 crystals. c) The composite *cas‐zz* (*cas‐zigzag*) chain, indicated by a red dashed box in b), projected along the *a*‐axis. This chain, whose periodicity is 5.1 Å, is built from the *cas* ([5^4^.6^2^]) composite building units that are stacked to form a chain. The *cas* chain further connects to a *zigzag* chain on each side to form a composite *cas‐zigzag* chain. d) Arrangements S and D, indicated by black dashed boxes in (b), projected along the *c*‐axis. These two arrangements were employed to describe that the two most adjacent *d5r* columns are at the same (S) height and at different (D) heights, respectively. e) Arrangements S and D projected along the [101] direction. Arrangement S creates the 10‐ring apertures perpendicular to the *b*‐axis, whereas the opposite holds for arrangement D. Thus, they can be regarded as open and closed valves that connect and block the 10‐ring channels, respectively. f) Arrangement D in the third direction. The pockets, which are formed instead the additional channels, are observable, and one of them is marked in red. g) One example of the channel system in the real structures of PST‐24. The 10‐ring channels along [101] by randomly distributed D arrangements are marked in light blue.

The average structure of PST‐24 has 11 symmetry‐independent tetrahedral sites (T‐sites). Its framework is built from [5^4^.6^2^] (*cas*) and [4^5^.5^2^] (double 5‐ring; *d5r*) units and *zigzag* chains (Figure 2), typical building units for zeolites. The *cas* units are stacked along the *b*‐axis and connected to a *zigzag* chain on each side to form a composite *cas‐zigzag* (*cas*‐*zz*) chain (Figure 2 c). The *cas‐zz* chains (shown in yellow) are isolated from one another by *d5r* columns (blue) (Figure 2 a–c). We note here that while the *cas‐zz* chains are periodically distributed in PST‐24 crystals, the two most adjacent *d5r* units in the average structure are too close to each other and cannot exist simultaneously. The only possibility of constructing the fully four‐connected framework with a reasonable bond geometry is to allow only every second *d5r* unit in each *d5r* column. In such a way, the *d5r* column along the *b*‐axis has a periodicity twice that of the *cas‐zz* chain, that is, the *b*‐parameter in a real crystal should be doubled (ca. 10.1696 Å). The sharp diffraction spots observed in the (*h* 0 *l*), (*h* ±2 *l*)… layers (Figure 1 b; Supporting Information, Figure S5a) also indicate that there are two possible arrangements for each *d5r* column, which are related by 1/2*b* Å (=5.0848(2) Å). The diffuse scattering in the (*h*±1 *l*), (*h*±3 *l*)… layers (Figure 1 b; Supporting Information, Figure S5b) extends in both *a*‐ and *c*‐axes, implying that the disorder is 2D. This means that each *d5r* column can adopt any of the two arrangements, and the choice is independent of other *d5r* columns. Therefore, it can be concluded that the 3D framework of PST‐24 is composed of two distinct types of regions on the molecular scale: *cas‐zz* chains that are ordered throughout the crystal and *d5r* columns that are disordered in nature. While each *cas‐zz* chain connects four *d5r* columns, each *d5r* column links two *cas‐zz* chains. Regardless of the presence of the disorder, in consequence, PST‐24 has at least a 2D pore system, with parallel straight 10‐ring (5.8×5.4 Å) and 8‐ring (4.8×3.1 Å) channels along the *b*‐axis (Figure 2 b) and sinusoidal 8‐ring channels along the *c*‐axis (Figure 2 g).

On the other hand, the two most adjacent *d5r* columns are connected by a 6‐ring (marked in Figure 2 b). The *d5r* column pair can adopt two possible arrangements; either at the same height *b* or at different heights related by 1/2*b* shift. Here we define these two *d5r* column arrangements as arrangements S (same) and D (different), respectively (Figure 2 d). It should be noted that arrangement S creates 10‐ring apertures (6.1×3.5 Å) to connect channels running along the *c*‐axis, whereas arrangement D blocks the connection between those channels. This leads to the formation of a pocket, but which is still part of sinusoidal 8‐ring channels (Figure 2 e–g). Therefore, arrangements S and D can be metaphorically considered as the open and closed states of a two‐way valve, respectively. In actual PST‐24 crystals, arrangements S and D are distributed randomly along the *a*‐ and *c*‐axes (Figure 2 g). As a result, PST‐24 adapts varying intracrystalline channel dimensionality, from 2D in arrangement D to 3D in arrangement S. Indeed, random changes in the dimensionality within real PST‐24 crystals should then cause notable differences in the intracrystalline molecular diffusion. Such a combination of ordered and disordered chain regions renders PST‐24 structurally very unique.

To further confirm the real PST‐24 structure model with a random distribution of arrangements S and D, we performed a simulation of ED and PXRD patterns using a model with a 10*a*×1*b*×10*c* (*b*=10.1696 Å) supercell that contains major features of the disorder in PST‐24 (see the Supporting Information for more details). Despite the limited size of the supercell for representing the complete disorder, the simulated ED and PXRD patterns match very well with the experimental ones, including the broad peak region in the PXRD pattern (Supporting Information, Figures S5 and S7), which confirms our model of this novel disordered structure.

**Figure 3:**
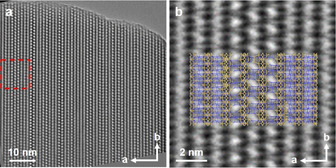
a) Structure projection of calcined, pure‐silica PST‐24 reconstructed from 17 through‐focus HRTEM images taken along the *c*‐axis. b) Enlarged image of the area marked in the red box in (a), which is superimposed with the structure model in order to reveal the local disorder in the image.

HRTEM also supports that PST‐24 crystals are 1D ordered (along the *b*‐axis) and 2D disordered (along the *a*‐ and *c*‐axes). The HRTEM image of Si‐PST‐24 (Figure 3) along the *c*‐axis shows periodic features along the *b*‐axis, corresponding to the *cas‐zz* chains and *d5r* columns (Figure 3 b). Features with the brightest contrast correspond to the sinusoidal 8‐ring channels where the majority of *d5r* columns along the *c*‐axis are at the same height (ordered). Depending on the height of *d*5*r* columns, the 8‐ring channels can have different orientations as shown in Figure 3 b. When the *d*5*r* columns along the *c*‐axis are at different heights, the 8‐ring channels become more sinusoidal and are less observable in the projection. The disorder in the *a* direction is more obvious with large variations of the contrasts, which is caused by different ratios of the *d5r* column arrangements in the projection (Figure 3). HRTEM also shows that the arrangement of *d5r* columns is quite random along both *a*‐ and *c*‐axes. This explains why the crystal in the HRTEM image is heavily disordered so that the ordered regions are limited to only a few atomic columns.

The average structure of as‐made Si‐PST‐24 was also solved and refined against the cRED data (Supporting Information, Table S1).^13^ While the same framework as that of calcined Si‐PST‐24 was obtained, the difference Fourier map of the refined average framework showed relatively large residual electron densities within the *d5r* units with half occupancy compared to the other regions (Supporting Information, Figure S8). Therefore, the PMI^+^ ions appears to be alternate with the *d5r* units along the *d5r* column in actual crystals. In fact, we were successful to energy‐minimize the configuration of PMI^+^ ions within the sinusoidal 8‐ring channel along the *c*‐axis (Supporting Information, Figure S9).

**Figure 4:**
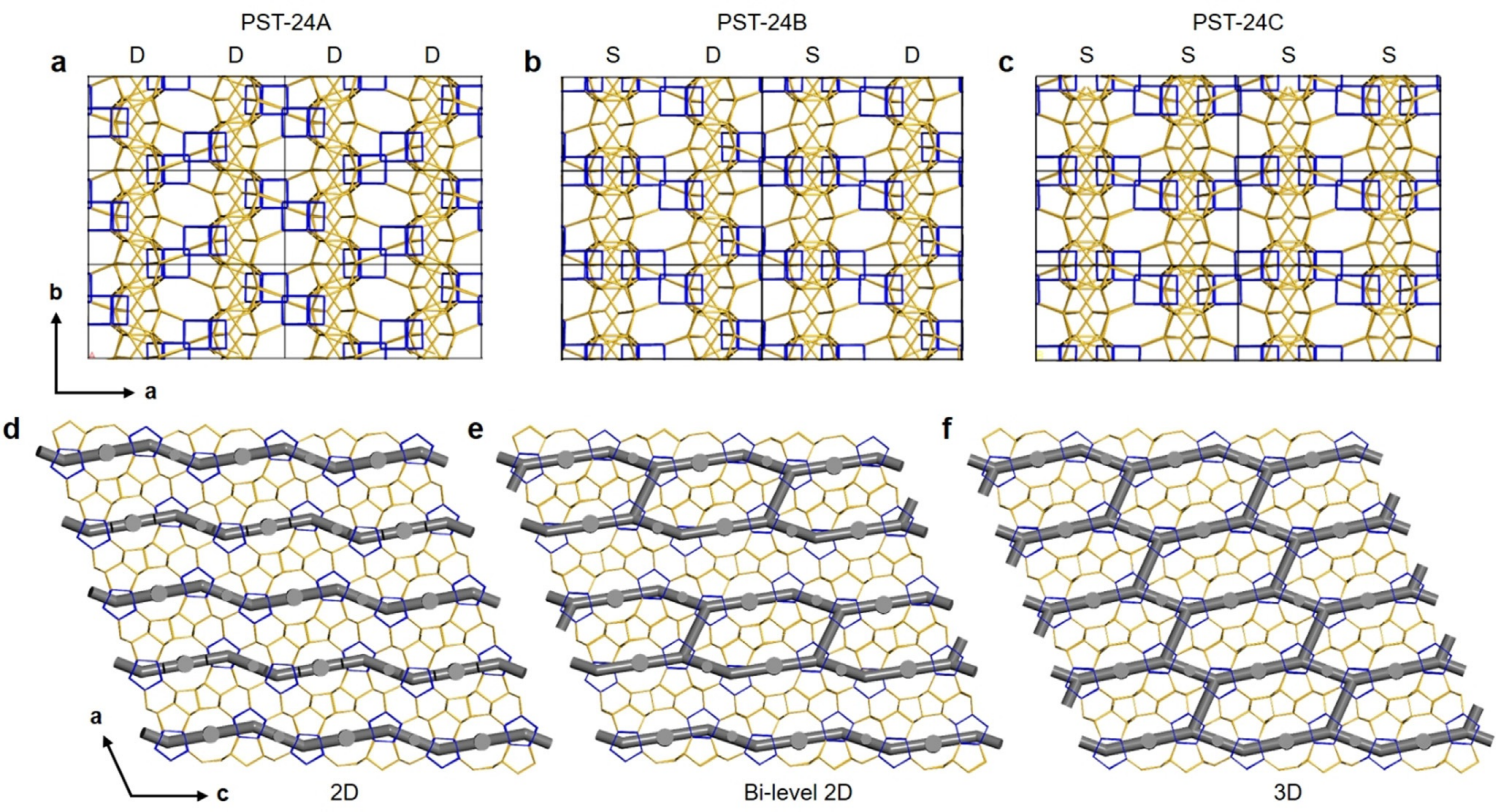
a)–c) Structural models of three polytypes PST‐24A, PST‐24B, and PST‐24C viewed along the *c*‐axis, showing arrangements DDDD…, SDSD…, and SSSS… along the *a*‐axis, respectively. Their corresponding space groups are *P*2_1_/*c*, *P* $\bar{1}$ , and *P*2/*c*, respectively. The *cas‐zz* chains are colored in yellow, and the *d5r* units in blue. Only the Si−Si connections are shown for clarity. d)–f) The channel systems in the three polytypes, showing differences in the channel dimensionality: 2D in PST‐24A; bi‐level 2D in PST‐24B; 3D in PST‐24C.

In principle, infinite numbers of ordered structures could be predicted by simply manipulating the arrangements of *d5r* columns. However, within the given *a* and *c* unit cell parameters, we were able to construct three ordered PST‐24 polytypes with: 1) arrangement D only, 2) an alternation of arrangements D and S along the *a*‐axis, and 3) arrangement S only, denoted as PST‐24A (*P*2_1_/*c*), PST‐24B (*P* $\bar{1}$ ), and PST‐24C (*P*2/*c*), respectively (Figure 4; Supporting Information, Figure S10 and Table S6). Of particular interest is that although the channels along the *b*‐ and *c*‐axes are almost identical to one another, they have different channel dimensionalities, depending on the status of the valves. PST‐24A with arrangement D has closed valves only, resulting in a 2D channel system (Figure 4 d). In PST‐24B with an alternation of the two types of arrangements, half of the valves are open to connect the adjacent channels along the *c*‐axis, forming a bi‐level 2D channel system (Figure 4 e). Finally, PST‐24C with arrangement S, where all valves are open, should possess a 3D channel system (Figure 4 f). To our knowledge, therefore, PST‐24 is the first example of disordered zeolites whose polytypes have similar overall pore structures but different channel dimensionalities. When the framework structures of the three polytypes in the pure‐silica form are optimized using the Sanders–Leslie–Catlow potential with *P*1 symmetry, the corresponding framework energies relative to α‐quartz were calculated to be similar to one another (11.6, 11.8, and 11.7 kJ (mol Si)^−1^, respectively).^14^ This indicates no preference to a particular polytype, explaining the variation of channel dimensionality in actual PST‐24. Their structures were also determined to have the same framework density (18.7; defined as the number of T‐atoms per 1000 Å^3^).

Apart from the disorder of *d5r* units, finding this unique type of building units in the zeolite structure is of much interest because the medium‐pore borosilicate (Si/B≈30) zeolite SSZ‐58 synthesized using 1‐butyl‐1‐cyclooctylpyrrolidinium cation as an OSDA in hydroxide media is the only prior case where the *d5r* unit has been observed.^15^ Considering that PST‐24 is essentially pure‐silica, the occurrence of *d5r* units during zeolite crystallization is in principle possible without the aid of heteroatoms other than Si. On the other hand, the ^19^F MAS NMR spectrum of as‐made Si‐PST‐24 shows a prominent resonance at −64 ppm (Supporting Information, Figure S11). This led us to first suspect the encapsulation of F^−^ ions within its *d5r* units. While the 6‐hedral ([4.5^2^.6^2^]) *t‐mel* units also exist in the PST‐24 structure, however, as‐made, pure‐silica ZSM‐5 was reported to have a ^19^F resonance at the same chemical shift, due to the encapsulation of F^−^ ions within *t‐mel* units, which has also been confirmed by single‐crystal X‐ray crystallography.^16^ Further study is in progress to accurately locate the F^−^ ions in as‐made Si‐PST‐24.

**Figure 5:**
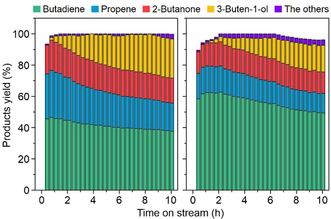
Yields of products in 1,3‐butanediol dehydration at 300 °C and 1.4 h^−1^ WHSV over H‐ZSM‐5 (left) and H‐Al‐PST‐24 (right). The bulk Si/Al ratios of H‐ZSM‐5 and H‐Al‐PST‐24 are 95 and 200, respectively. A 10 wt % aqueous solution of 1,3‐butanediol was used as a feed.

While the dehydration of 1,3‐butanediol is of technological interest to produce butadiene, a major industrial chemical used mainly for the manufacture of synthetic rubbers and resins, high‐silica (Si/Al=130) H‐ZSM‐5 zeolite has recently been reported to be a promising catalyst for this dehydration at relatively low temperatures (<400 °C).^17^ Finally, we evaluated the catalytic properties of the proton form of Al‐PST‐24 (H‐Al‐PST‐24) with Si/Al=200, for 1,3‐butanediol dehydration at 300 °C and 1.4 h^−1^ weight hourly space velocity and compared the results with those of H‐ZSM‐5 with Si/Al=95 (Figure 5). The former catalyst is always characterized by a higher butadiene yield. In our view, the outstanding performance of H‐Al‐PST‐24 for this reaction can be attributed not only to its low acid site density (Supporting Information, Figure S12) but also to unique pore structure (Figure 2).

## Conclusion

We have synthesized a novel medium‐pore zeolite PST‐24 under high excess fluoride conditions (HF/PMIOH=3). cRED combined with HRTEM revealed both the structure and the unique *d5r* columnar disorder of PST‐24. The composite *cas‐zz* chains and *d5r* columns are arranged alternatively extending in two (*a* and *c*) directions and connected to form the 3D framework of PST‐24. While the *cas‐zz* chains are ordered throughout the entire crystal, the *d5r* column pairs can adopt two arrangements, which close and open 10‐ring channels, respectively. This arrangement leads to a unique channel system in actual PST‐24 crystals, varying locally from 2D to 3D, which can influence the intracrystalline diffusivity of guest molecules. Three ordered PST‐24 polytypes have been proposed based on the arrangements of *d5r* columns and are characterized by consecutive changes in the channel dimensionality: 2D, bi‐level 2D, and 3D. High‐silica (Si/Al=200) PST‐24 was found to be highly active and selective for the dehydration of 1,3‐butanediol to butadiene, mainly due to its unique pore architecture.

## Conflict of interest

The authors declare no conflict of interest.

## Supporting information

As a service to our authors and readers, this journal provides supporting information supplied by the authors. Such materials are peer reviewed and may be re‐organized for online delivery, but are not copy‐edited or typeset. Technical support issues arising from supporting information (other than missing files) should be addressed to the authors.

Supplementary
