# Supplementary material for: PST‐24: A Zeolite with Varying Intracrystalline Channel Dimensionality
Source: Angew Chem Int Ed Engl. 2020 Aug 11;59(40):17691–6. doi: 10.1002/anie.202007804 (PMC7540362; doi:10.1002/anie.202007804)
Supplement: Supplementary file 1 — Supplementary [file ANIE-59-17691-s001.pdf]

Angewandte Chemie
Eine Zeitschrift der Gesellschaft Deutscher Chemiker

Supporting Information

# PST-24: A Zeolite with Varying Intracrystalline Channel Dimensionality

*Donghui Jo+, Jingjing Zhao+, Jung Cho+, Jeong Hwan Lee, Yang Liu, Chang-jun Liu, Xiaodong Zou,* and Suk Bong Hong**

anie_202007804_sm_miscellaneous_information.pdf

## Experimental Section

### Zeolite Synthesis

The reagents, including pentamethylimidazolium hydroxide (PMIOH), were prepared and characterized as previously described.[S1] They are hydrofluoric acid (HF, 48% aqueous solution, J.T. Baker), tetraethylorthosilicate (TEOS, 98%, Aldrich), aluminum hydroxide ($Al(OH)_3 \cdot H_2O$, Aldrich), and deionized water. $PMI^+$-mediated synthesis of zeolites in fluoride media was carried out using synthesis mixtures with the chemical composition $0.5ROH \cdot xHF \cdot 1.0SiO_2 \cdot yAl_2O_3 \cdot 5.0H_2O$, where R is $PMI^+$ and $x$ and $y$ are varied between $0.25 \leq x \leq 2.0$ and $0 \leq y \leq 0.1$, respectively. In a typical synthesis, aluminum hydroxide was mixed with a solution of PMIOH and stirred at room temperature for 1 h. To this solution, a given amount of TEOS was added and the mixture was stirred at room temperature for 3 h. The resulting mixture was heated at 80 °C to remove ethanol molecules generated by the hydrolysis of TEOS and some water to obtain the desired composition. Then, an appropriate amount of HF was added while mixing with a spatula. The final synthesis mixture was transferred into a Teflon-lined 23 mL-autoclave and heated under rotation (60 rpm) at 175 °C for 14 days.

The solid products were recovered by filtration or centrifugation, washed repeatedly with water and dried overnight at room temperature. If required, as-made zeolites were converted into their proton form by calcination at 600 °C in air for 8 h. For comparison, the ammonium form of ZSM-5 with Si/Al = 95 were obtained from Tosoh and were converted into their proton form by twice refluxing in 1.0 M $NH_4NO_3$ solutions for 6 h followed by calcination at 550 °C in air for 2 h.

### Characterization

Powder X-ray diffraction (PXRD) patterns were recorded on a PANalytical X´Pert diffractometer (Cu Kα radiation) with an X´Celerator detector. Data were collected with a fixed divergence slit (0.50°) and Soller slits (incident and diffracted = 0.04 rad). Elemental analysis for Si and Al was carried out using a Shimadzu ICPE-9000 inductively coupled plasma spectrometer. The C, H, and N contents of the samples were analyzed by using a Vario EL III elemental organic analyzer. Thermogravimetric analyses (TGA) were performed in air on an SII EXSTAR 6000 thermal analyzer, where the weight losses related to the combustion of OSDAs were further confirmed by differential analyses (DTA) using the same analyzer. Crystal morphology and average size were determined using a JEOL JSM-6510 scanning electron microscopy (SEM). $N_2$ sorption experiments were carried out on a Mirae SI nanoPorosity-XG analyzer. $NH_3$ temperature-programmed desorption (TPD) was carried out on a fixed bed, flow-type apparatus linked to a Hewlett-Packard 5890 series II gas chromatograph with a thermal conductivity detector. A sample of ca. 0.1 g was activated in flowing He (50 mL $min^{-1}$) at 550 °C for 2 h. Then, 10% $NH_3$ was passed over the sample at 150 °C for 0.5 h. The treated sample was subsequently purged with He at the same temperature for 1 h to remove physisorbed $NH_3$. Finally, the TPD was performed in flowing He (30 mL $min^{-1}$) from 150 to 750 °C at a temperature ramp of 10 °C $min^{-1}$.

$^1H$ and $^{13}C$ solution nuclear magnetic resonance (NMR) measurements on the iodide form of the $PMI^+$ cation were performed in 5 mm quartz tubes using a Bruker AVANCE III 300 spectrometer. The $^1H$ NMR spectra were measured at a $^1H$ frequency of 300.13 MHz with a $\pi/2$ rad pulse length of 11.0 μs and a recycle delay of 2.0 s. The $^{13}C$ NMR spectra were recorded at a

$^{13}C$ frequency of 75.475 MHz with a π/2 rad pulse length of 10.2 μs and a recycle delay of 1.5 s. To measure $^{13}C$ solution NMR spectrum of the organic structure-directing agents (OSDAs) liberated from as-made PST-24, we dissolved the as-made zeolite of 0.5 g in HF (48% aqueous solution, 1.3 mL) and diluted the solution with 2 mL of $D_2O$.[S2] Solid-state multinuclear NMR measurements were carried out using a Bruker AVANCE III 500 HD spectrometer. The $^{19}F$ magic-angle-spinning (MAS) NMR spectra were obtained at a spinning rate of 10 kHz, a $^{19}F$ frequency of 470.54 MHz with a π/2 rad pulse length of 2.0 μs, a recycle delay of 2.0 s, and an acquisition of 256 pulse transients. The $^{19}F$ chemical shifts are referenced relative to $CFCl_3$. The $^{1}H$-$^{13}C$ cross polarization (CP) MAS NMR spectra were measured at a spinning rate of 20 kHz, a $^{13}C$ frequency of 125.79 MHz with a π/2 rad pulse length of 1.8 μs, a contact time of 2.0 ms, a recycle delay of 5.0 s, and an acquisition of ca. 7000 pulse transients. The $^{29}Si$ MAS NMR spectra were recorded at a spinning rate of 20 kHz, a $^{29}Si$ frequency of 99.377 MHz with a π/2 rad pulse length of 4 μs, recycle delay of 150 and 300 s for as-made and calcined PST-24 zeolites, respectively. Approximately 500 pulse transients were accumulated. The $^{13}C$ and $^{29}Si$ shifts are referenced relative to TMS.

## Structural Analysis

Both as-made and calcined PST-24 samples were investigated by TEM. The samples were crushed in a mortar and dispersed in ethanol. The suspension was then treated by ultrasonication for ca. 30 s. Three droplets from the suspension were applied on a lacey carbon TEM grid (Okenshoji). Continuous rotation electron diffraction (cRED) data were collected on a 200 kV JEOL JEM-2100 $LaB_6$ TEM at room temperature. The spot size and camera length were 3 and 30 cm, respectively. Video frames of selected area electron diffraction patterns were recorded by a high-speed hybrid camera (Timpix Quad). The rotation speed of the goniometer was 0.23° $s^{-1}$ and the exposure time was 0.5 s, covering 0.46° per frame. cRED data were collected from different crystals, and the total rotation angle of each data sets ranged between 70° and 115°. The cRED data were first processed using the software REDp[S3,S4] for the initial determination of unit cell parameters and space group. Then, the cRED data were processed by the program package XDS[S5], where the instrumental parameters (rotation axis, beam position, and beam orientation), unit cell, orientation matrix, and intensity profiles were refined, and the intensities were integrated. Data sets from different crystals were merged together by XSCALE in XDS. The resulting reflection list $|F(hkl)|^2$ was used by the program SHELX[S6] for structure solution and refinement. The unit cell parameters refined using the PXRD data are more accurate than those from cRED data, and were therefore used in the refinement against cRED data. Based on the reflection conditions, three possible space groups are deduced, *C*2 (No. 5), *Cm* (No. 8), and *C*2/*m* (No. 12). Using space groups *C*2 and *C*2/*m*, it was possible to solve the average structure from the cRED data by direct methods using the program SHELXT.[S6] Further structure refinement against cRED data merged from three crystals shows that *C*2/*m* gave a framework model with the best bond geometry. In order to take into account the disorder of the double 5-ring (*d*5*r*) columns during the refinement, the atoms belonging to the *d*5*r* unit are with an occupancy of 0.5. Soft restraints were applied on the Si-O (1.61 Å) and O…O distances (2.62 Å). Basic experimental parameters for cRED data collection, crystallographic data and detailed refinement results are given in Table S1.

A through-focus[S7] series of 17 HRTEM images were recorded on a Gatan 2k × 2k charge-coupled device camera on a JEOL-2100F TEM at 200 kV. The focus step was -53.3 Å and the exposure time was 5 s per image. The structure projection reconstruction was performed using the

software QFocus[S8]. The starting defocus was determined to be -183 Å and the twofold astigmatism to be 188 Å with azimuth angles of 117°.

Synchrotron PXRD data for PST-24 were collected on the 9B beamline equipped with a ceramic furnace at the Pohang Acceleration Laboratory (PAL; Pohang, Korea) using monochromated X-rays ($\lambda$ = 1.5175 Å). The detector arm of the vertical scan diffractometer consists of seven sets of Soller slits, flat Ge(111) crystal analyzers, anti-scatter baffles, and scintillation detectors, with each set separated by 20°. Data were obtained on the sample at room temperature in flat plate mode, with a step size of 0.02° and overlaps of 0.5° to the next detector bank. The fractional coordinates of the PST-24 structure obtained from cRED analysis were refined along with the unit cell and instrumental parameters against the synchrotron PXRD data, via the Rietveld method[S9] using the GSAS suite of programs[S10] and EXPGUI graphical interface,[S11] while excluding the region of broad diffraction peaks ($2\theta$ < 12°). During the refinements, the framework Si-O and O…O distances were soft-constrained to 1.60 Å ($\sigma$ = 0.05 Å) and 2.62 Å ($\sigma$ = 0.05 Å), respectively.[S12] Peak shape was modeled using the pseudo-Voigt profile function.[S13] The isotropic atomic displacement parameters of the framework atoms have been constrained in groups, according to the type of atoms (Si or O) and occupancy (1 or 0.5). The convergence was achieved by refining simultaneously all profile parameters, scale factor, lattice constants, $2\theta$ zero-point, and atomic positions, thermal displacement parameters, and occupancy factors for the framework atoms. The data collection and crystallographic parameters are summarized in Tables S2-S5. The final Rietveld plot, which is displayed in Figure S6, provides a reasonable match between the observed and simulated PXRD patterns. The dimensions of the pore opening are calculated using an oxygen radius of 1.35 Å.

Three different polytypes of PST-24 were built from the refined average structure, and their symmetries were identified by the 'Find Symmetry' tool implemented in the Materials Studio[S14] (Figure S10 and Table S6). Their framework energies relative to $\alpha$-quartz[S15] were calculated using the Sanders-Leslie-Catlow potential[S16] in the GULP program[S17].

**Simulation of ED and PXRD Patterns Using a Supercell**

An in-house script was written to generate a supercell model with a random distribution of each disorder type. The script, which is available on github (chojy8300), can read atomic coordinates from a crystallographic information file (cif) and require each unit to be written as a separate file. All *d5r* coordinates were output as cifs that were then distributed throughout the structure with a Monte Carlo algorithm to have a uniform distribution of arrangements S and D. The code can be easily modified to generate a desired supercell dimension. For this work, a $10a \times 1b \times 10c$ supercell ($b$ = 10.1696 Å) of calcined PST-24 was constructed automatically, while utilizing the DLS76 optimized atomic coordinates. The simulated kinematical diffraction intensities of the ED and PXRD patterns were generated using the supercell by CrystDiff[S18] and Materials Studio[S14], respectively (Figures S5e,f and S7).

**Molecular Modeling of Intrazeolitic OSDAs**

A free $PMI^+$ ion was optimized using DMol3 with PW91 generalized gradient approximation exchange-correlation function convergent within $10^{-4}$ Ha, as implemented in Material Studio. Then, the $PMI^+$ ions were manually introduced in an ordered PST-24 structure (polytype PST-24A) so that themselves alternate with the *d5r* units along the *d5r* columns while accounting for chemical

sensibility. The configuration of the $PMI^+$ ions was optimized using Dreiding force field[S19] in the GULP program (Figure S9). Only dispersion contributions were assessed.

## Catalysis

Catalytic experiments were conducted at atmospheric pressure in a continuous-flow apparatus with a fixed-bed microreactor. The dehydration of 1,3-butanediol was conducted at 300 °C with a weight hourly space velocity of 1.4 $h^{-1}$. Before the experiments, each catalyst was pretreated under $N_2$ flow (50 mL $min^{-1}$) at 500 °C for 2 h and kept at 300 °C to define a standard operating procedure, allowing time for the reactant/carrier gas distribution to stabilize. A reactant stream of 10 wt% aqueous solution of 1,3-butanediol (99%, Alfa) was then fed at a rate of 1.4 mL $h^{-1}$ into the reactor containing 0.1 g of the catalyst. The total gas flow at the reactor inlet was kept constant at 30 mL $min^{-1}$. The reaction products were analyzed online in an Agilent 7890A gas chromatograph equipped with a HP-5 capillary column (30 m × 0.25 mm) and an FID, with the first analysis carried out after 30 min on stream. The conversion of 1,3-butanediol was defined as the percentage of 1,3-butanediol consumed during the reaction, and the yield of each product was calculated as the percentage of the amount of 1,3-butnaediol converted to reaction products.

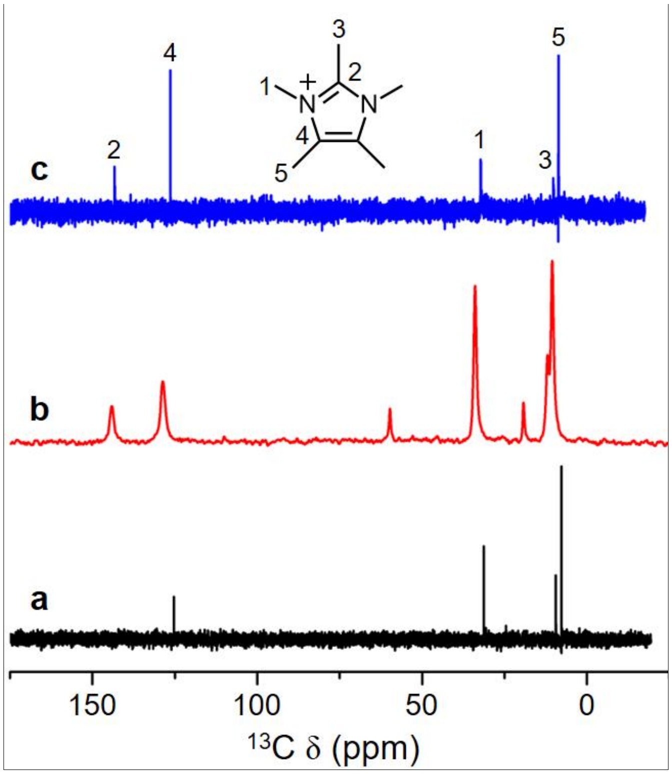


**Figure S1.** $^{13}$C NMR spectra of OSDA, PMI$^+$: a) $^{13}$C NMR of a $D_2O$ solution of the iodide form of PMI$^+$, b) $^1$H-$^{13}$C CP MAS NMR of as-made, pure-silica PST-24 with PMI$^+$ occluded in the pores, and c) $^{13}$C NMR of as-made PST-24 dissolved in HF. These data reveal that the $^{13}$C resonances around 19 and 60 ppm (indicated by arrows) in the $^1$H-$^{13}$C CP MAS NMR spectrum of as-made PST-24 may also originate from the occluded organic species rather than from the decomposed ones. It should be noted that the resonance due to the imidazolium ring carbon atom at position 2 in PMI$^+$ is not observed in the solution spectrum a), probably because of the reduced nuclear Overhauser enhancement. The appearance of this resonance in the solution spectrum c) seems to be due to the low pH of the solution of HF-dissolved as-made PST-24.

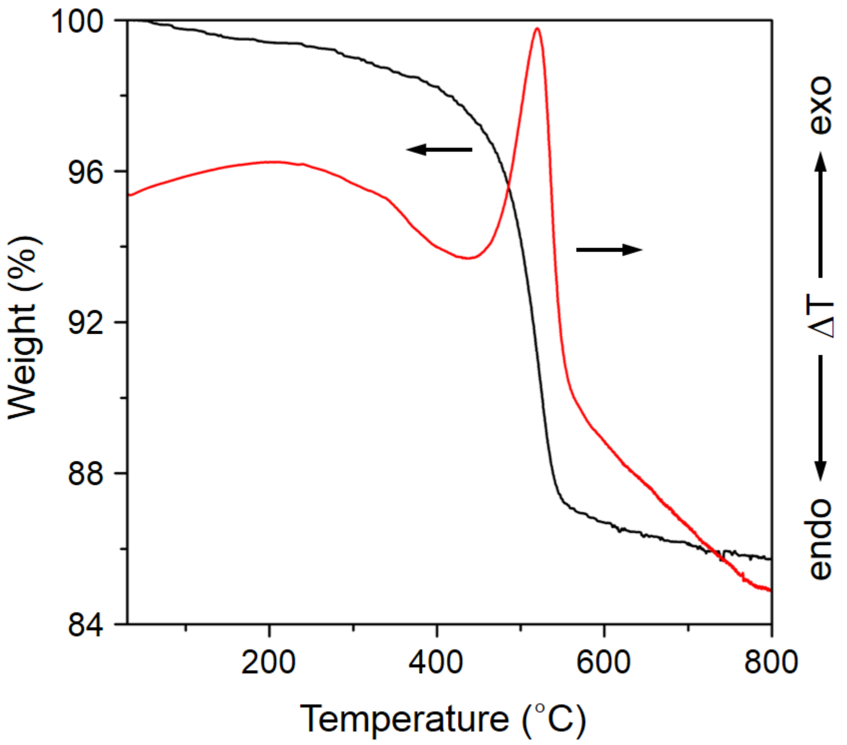


**Figure S2.** TGA/DTA profiles of as-made, pure-silica PST-24.

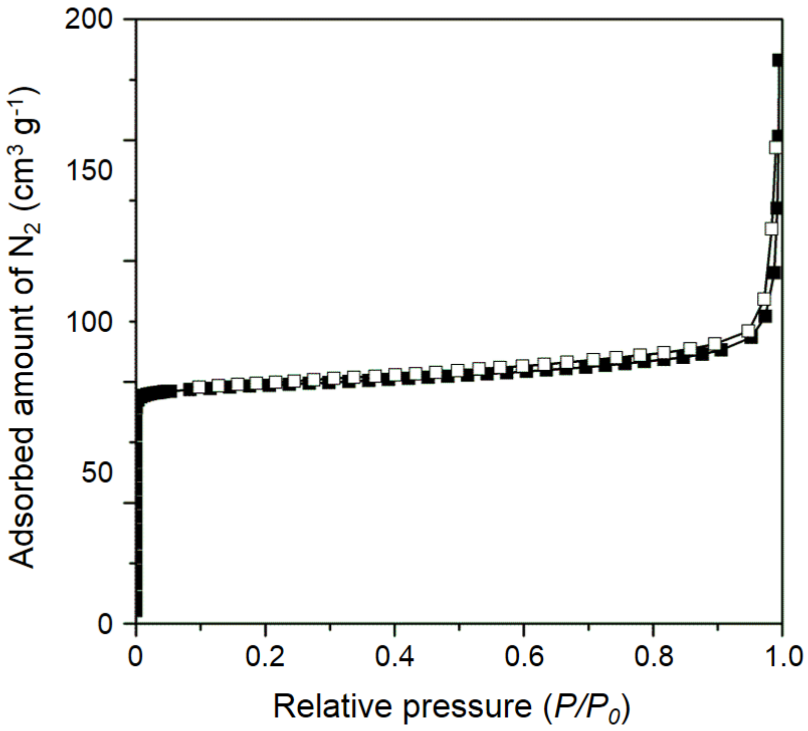


**Figure S3.** $N_2$ adsorption (closed) and desorption (open) isotherm of the calcined, pure-silica PST-24. Its BET surface area and micropore volume were calculated to be 390 (microporous, 360; external, 30) $m^2$ $g^{-1}$ and 0.14 $cm^3$ $g^{-1}$, respectively.

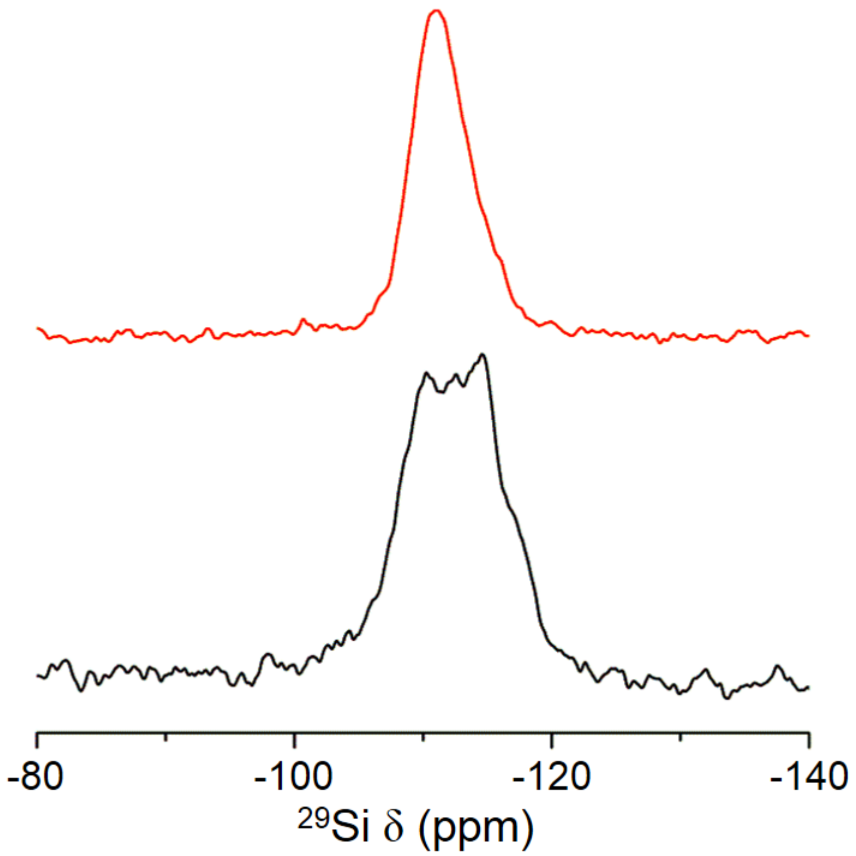


**Figure S4.** $^{29}Si$ MAS NMR spectra of the as-made (black) and calcined (red) forms of pure-silica PST-24.

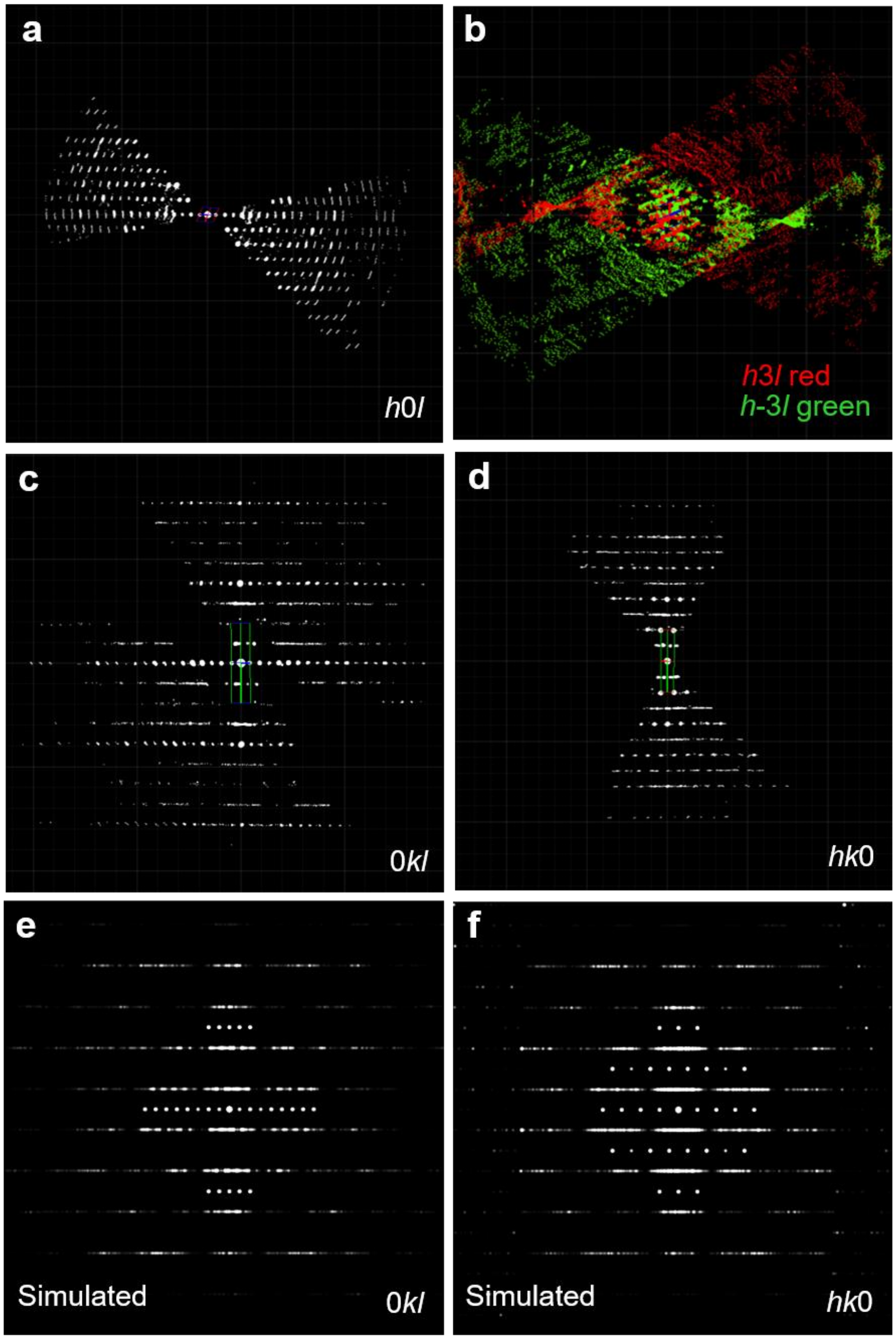


**Figure S5.** a)-d) 2D slices of *h*0*l*, *h*±3*l*, 0*kl*, and *hk*0 reciprocal lattice planes reconstructed from the cRED data of calcined, pure-silica PST-24. The *h*3*l* and *h*-3*l* planes show that the diffuse scattering is quite random without any specific rules. e), f) Simulated 0*kl* and *hk*0 diffraction patterns by CrystDiff from a 10*a* × 1*b* × 10*c* supercell containing a random distribution of arrangements S and D. The supercell was generated by a Monte Carlo algorithm. The experimental and simulated diffraction patterns show a good agreement with each other, despite the limited size of the supercell used for this simulation.

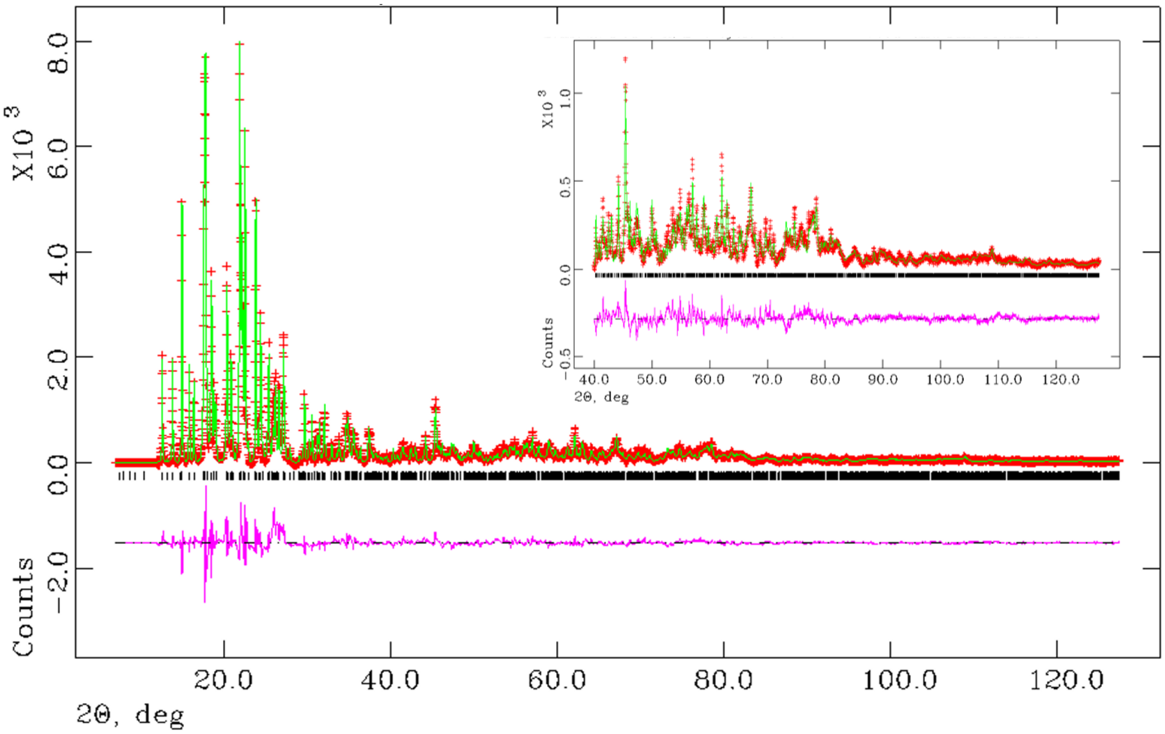


**Figure S6.** Rietveld plot of calcined, pure-silica PST-24 (λ = 1.5175 Å; 2$\theta$ ≥ 12°): observed data (+), calculated fit (solid line), and difference plot (lower trace). The tick marks represent the positions of allowed reflections.

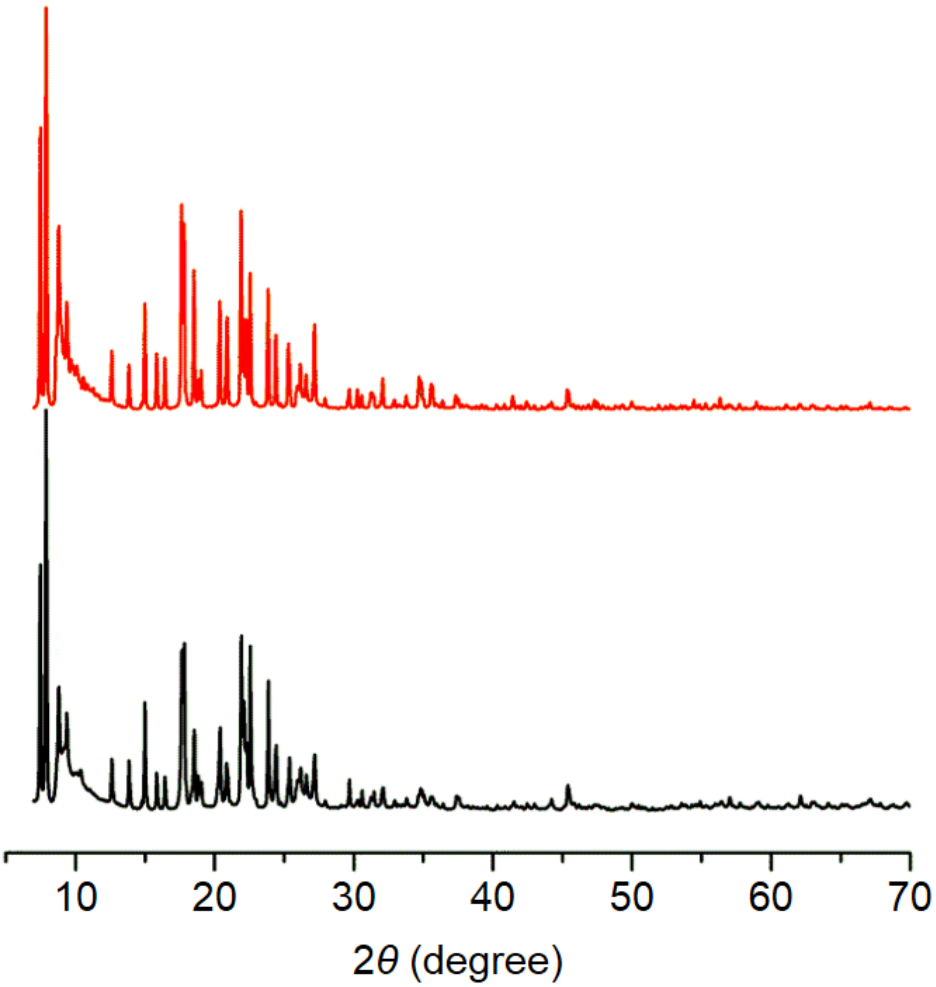


**Figure S7.** The experimental (black) and simulated (red) PXRD patterns of calcined, pure-silica PST-24 ($\lambda$ = 1.5175 Å). The simulation was carried out by Materials Studio from a 10$a$ × 1$b$ × 10$c$ supercell.

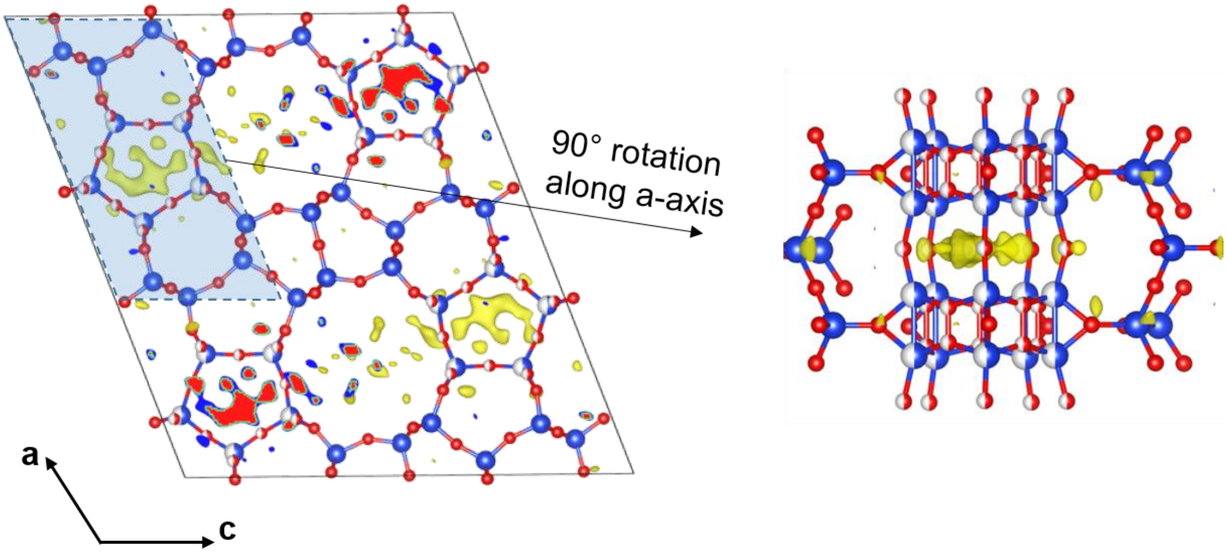


**Figure S8.** Difference Fourier map ($F_o$ - $F_c$) of average structure of as-made, pure-silica PST-24 refined against the cRED data. The positive map (yellow) was countered at the isosurface level of 0.45.

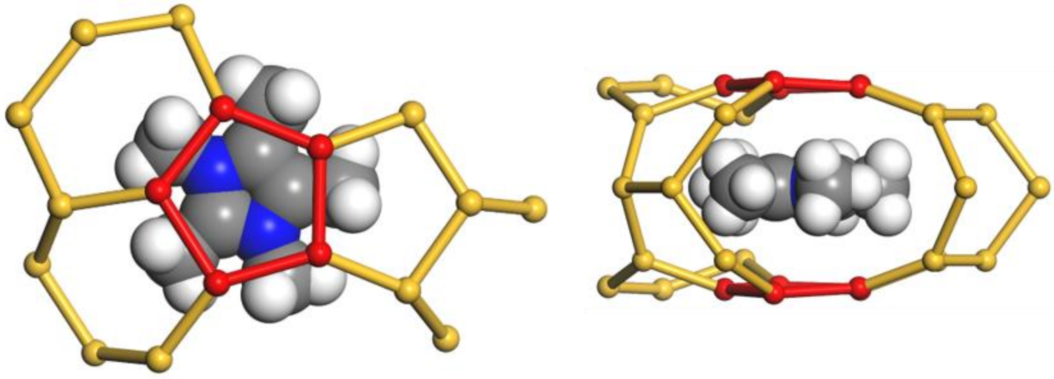

**Figure S9.** Energy-minimized configuration of the PMI$^+$ ion within the sinusoidal 8-ring channel along the *c*-axis of PST-24A. The 5-rings belonging to *d5r* units are marked in red.

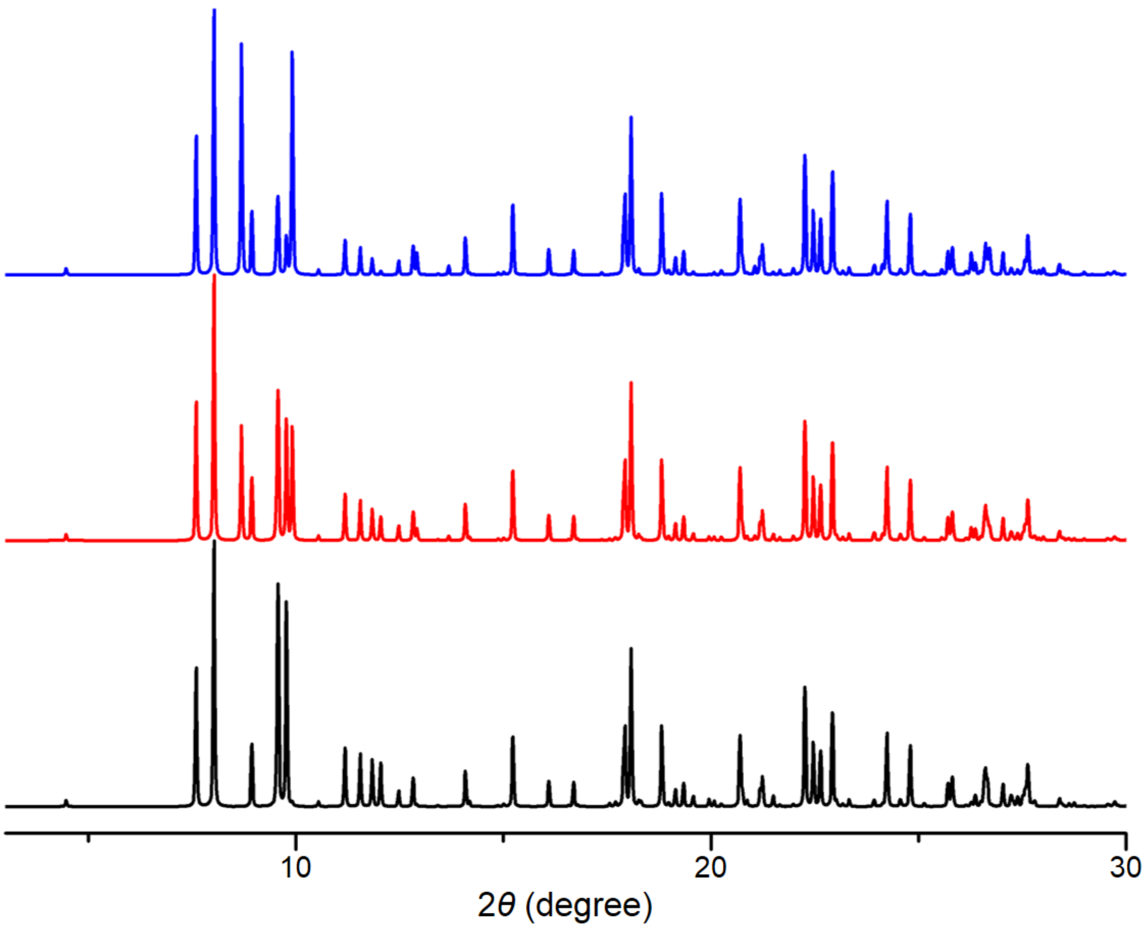


**Figure S10.** Simulated PXRD patterns of three PST-24 polytypes: (from bottom to top) PST-24A, PST-24B, and PST-24C (λ = 1.54056 Å).

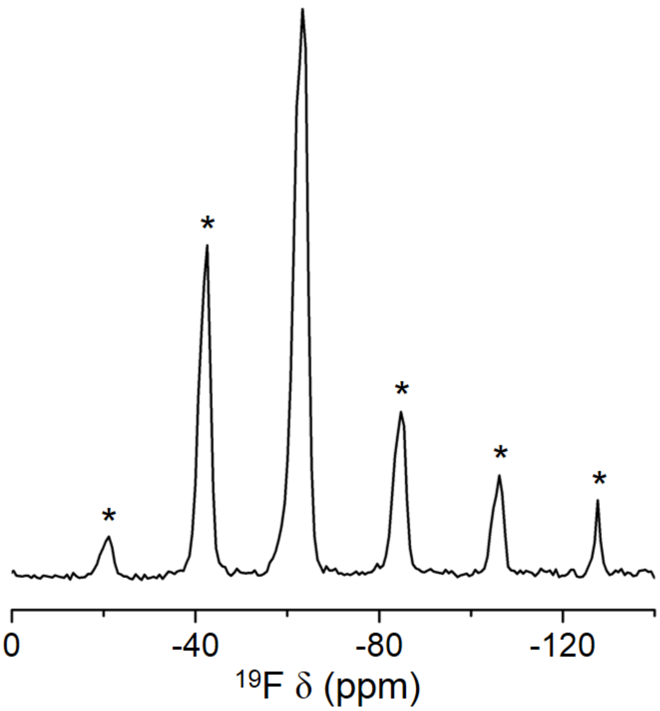


**Figure S11.** $^{19}F$ MAS NMR spectrum of as-made, pure-silica PST-24. Spinning side bands are marked by asterisks.

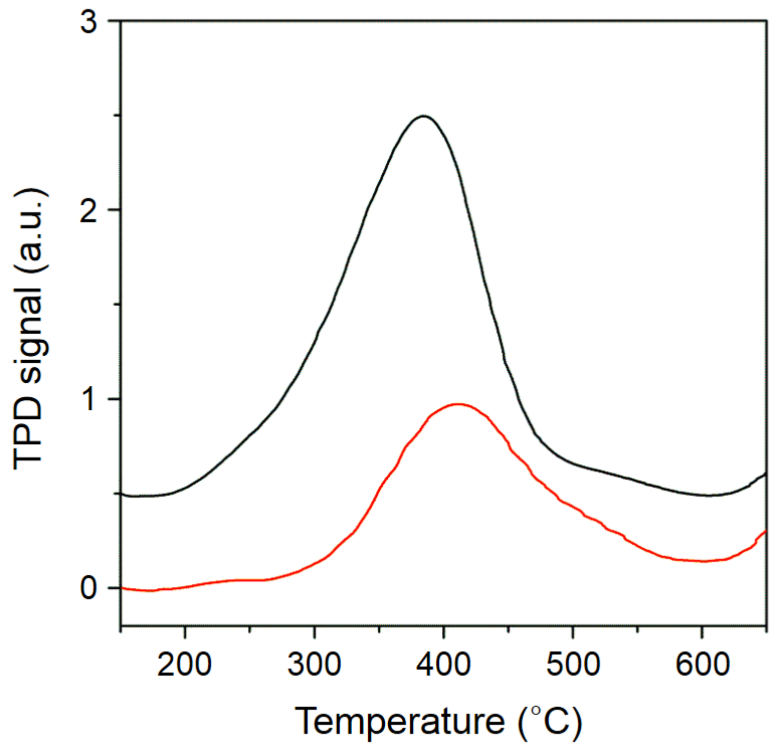


**Figure S12.** $NH_3$ TPD profiles for H-ZSM-5 (black) and H-Al-PST-24 (red). The bulk Si/Al ratio of each zeolite is 95 and 200, respectively.

**Table S1.** Experimental parameters for cRED data collection and crystallographic information for the averaged structures of the as-made and calcined forms of pure-silica PST-24.[a]

| Sample | As-made PST-24 | Calcined PST-24 |
|---|---|---|
| Wavelength (Å) | 0.0251 | 0.0251 |
| Rotation range (°) | 70-115 | 70-110 |
| Rotation speed (°/s) | 0.23 | 0.23 |
| Exposure time/frame (s) | 0.5 | 0.5 |
| No. of data sets | 7 | 3 |
| Crystal width (nm) | 100-200 | 100-200 |
| Resolution (Å) | 0.90 | 0.90 |
| Crystal system | Monoclinic | Monoclinic |
| Space group | *C*2/*m* (No. 12) | *C*2/*m* (No. 12) |
| Unit cell parameters (cRED) | | |
| *a* (Å) | 24.313(44) | 24.140(44) |
| *b* (Å) | 5.384(2) | 5.211(18) |
| *c* (Å) | 22.016(39) | 21.761(30) |
| $\beta$ (°) | 111.422(190) | 111.389(180) |
| Completeness | 0.846 | 0.918 |
| Total reflections | 12752 | 7374 |
| $R_{int}$ | 0.2583 | 0.1797 |
| No. unique reflections $I > 2\sigma(I)$/all | 1391/1642 | 1469/1788 |
| No. of parameters | 113 | 111 |
| No. of restrains | 57 | 87 |
| $R_1(F > 4\sigma(F))/R_1(\text{all})$ | 0.2148/0.2387 | 0.3387/0.3684 |
| GOF | 2.102 | 3.072 |

[a] The unit cell parameters, obtained by the Pawley and Rietveld (see Table S2) fits of the as-made (lab) and calcined (synchrotron) PXRD patterns, respectively, were used for the structure refinement. The unit cell parameters from the cRED data are slightly larger than those from the PXRD data, presumably due to lens distortions and the sample height changing during the data collection.

**Table S2.** Crystallographic and experimental parameters for the Rietveld refinement of calcined, pure-silica PST-24.

| | |
|---|---|
| Unit cell composition | $\|(H_2O)_{1.6}\|[Si_{44}O_{88}]$ |
| Refined structure | $[Si_{44}O_{88}]$ |
| Crystal system | Monoclinic |
| Space group | *C*2/*m* (No. 12) |
| *a* (Å) | 23.7125(6) |
| *b* (Å) | 5.0848(2) |
| *c* (Å) | 21.3024(6) |
| $\beta$ (°) | 111.838(2) |
| Unit cell volume (Å$^3$) | 2384.2(1) |
| X-ray source | 9B beamline, PAL |
| Wavelength (Å) | 1.5175 |
| Step size (°) | 0.02 |
| $2\theta$ scan range (°) | 7-127.5 |
| No. of contributing reflections | 2298 |
| No. of restraints | 118 |
| No. of parameters | 117 |
| $R_{wp}$ (%) | 9.17 |
| $R_F^2$ (%) | 11.1 |
| $\chi^2$ | 6.59 |

**Table S3.** Atomic coordinates and thermal parameters for calcined, pure-silica PST-24 refined by the Rietveld method.

| Atom | *x* | *y* | *z* | Occupancy | $U_{iso}$ (× 100 Å$^2$) | Multiplicity |
|---|---|---|---|---|---|---|
| Si1 | 0.42429(32) | 0 | 0.0972(4) | 1 | 1.66(5) | 4 |
| Si2 | 0.38123(32) | 0.5 | 0.3996(4) | 1 | 1.66(5) | 4 |
| Si3 | 0.46729(31) | 0.5 | 0.3188(4) | 1 | 1.66(5) | 4 |
| Si4 | 0.38746(32) | 0.5 | 0.16045(35) | 1 | 1.66(5) | 4 |
| Si5 | 0.56741(32) | 1 | 0.5073(4) | 1 | 1.66(5) | 4 |
| Si6 | 0.55024(32) | 1 | 0.3568(4) | 1 | 1.66(5) | 4 |
| Si7 | 0.55013(33) | 0.1962(15) | 0.1286(4) | 0.5 | 1.56(10) | 8 |
| Si8 | 0.6346(4) | 0.1877(15) | 0.0476(4) | 0.5 | 1.56(10) | 8 |
| Si9 | 0.76506(31) | 0.1945(14) | 0.1595(4) | 0.5 | 1.56(10) | 8 |
| Si10 | 0.26382(35) | 0.3024(15) | 0.3031(4) | 0.5 | 1.56(10) | 8 |
| Si11 | 0.12859(34) | 0.3023(15) | 0.2859(4) | 0.5 | 1.56(10) | 8 |
| O1 | 0.3995(4) | 0.7428(9) | 0.45164(31) | 1 | 3.51(13) | 8 |
| O2 | 0.40668(31) | 0.2433(9) | 0.13216(30) | 1 | 3.51(13) | 8 |
| O3 | 0.50489(28) | 0.7596(9) | 0.3301(4) | 1 | 3.51(13) | 8 |
| O4 | 0.3811(4) | 0 | 0.01741(25) | 1 | 3.51(13) | 4 |
| O5 | 0.81598(21) | 0 | 0.1490(4) | 1 | 3.51(13) | 4 |
| O6 | 0.49336(21) | 0 | 0.1085(5) | 1 | 3.51(13) | 4 |
| O7 | 0.4239(5) | 0.5 | 0.2407(4) | 1 | 3.51(13) | 4 |
| O8 | 0.31137(26) | 0.5 | 0.3556(4) | 1 | 3.51(13) | 4 |
| O9 | 0.42134(28) | 0.5 | 0.3552(4) | 1 | 3.51(13) | 4 |
| O10 | 0.60101(24) | 1 | 0.32584(26) | 1 | 3.51(13) | 4 |
| O11 | 0.5790(4) | 1 | 0.43870(27) | 1 | 3.51(13) | 4 |
| O12 | 0.5 | 1 | 0.5 | 1 | 3.51(13) | 2 |
| O13 | 0.70090(23) | 0.1182(18) | 0.10094(33) | 0.5 | 2.54(31) | 8 |
| O14 | 0.75636(30) | 0.0994(21) | 0.22656(21) | 0.5 | 2.54(31) | 8 |
| O15 | 0.19694(19) | 0.3830(18) | 0.3019(4) | 0.5 | 2.54(31) | 8 |
| O16 | 0.59584(35) | 0.1243(18) | 0.20537(23) | 0.5 | 2.54(31) | 8 |
| O17 | 0.59124(21) | 0.1085(18) | 0.08667(24) | 0.5 | 2.54(31) | 8 |
| O18 | 0.2774(7) | 0 | 0.3260(13) | 0.5 | 2.54(31) | 4 |
| O19 | 0.1217(6) | 0 | 0.2976(7) | 0.5 | 2.54(31) | 4 |
| O20 | 0.7799(6) | 0.5 | 0.1577(8) | 0.5 | 2.54(31) | 4 |
| O21 | 0.6310(7) | 0.5 | 0.0302(7) | 0.5 | 2.54(31) | 4 |
| O22 | 0.5335(6) | 0.5 | 0.1199(8) | 0.5 | 2.54(31) | 4 |

**Table S4.** Bond lengths for calcined, pure-silica PST-24 refined by the Rietveld method.

| | Bond length (Å) |
|---|---|
| Si1-O2 (×2) | 1.577(8) |
| Si1-O4 | 1.627(8) |
| Si1-O6 | 1.565(9) |
| Si2-O1 (×2) | 1.607(7) |
| Si2-O8 | 1.571(8) |
| Si2-O9 | 1.571(12) |
| Si3-O3 (×2) | 1.561(6) |
| Si3-O7 | 1.598(9) |
| Si3-O9 | 1.555(12) |
| Si4-O2 (×2) | 1.575(8) |
| Si4-O5 | 1.620(9) |
| Si4-O7 | 1.604(9) |
| Si5-O1 (×2) | 1.606(6) |
| Si5-O11 | 1.585(11) |
| Si5-O12 | 1.547(8) |
| Si6-O3 (×2) | 1.587(7) |
| Si6-O10 | 1.573(11) |
| Si6-O11 | 1.621(9) |
| Si7-O6 | 1.600(8) |
| Si7-O16 | 1.632(9) |
| Si7-O17 | 1.611(11) |
| Si7-O22 | 1.588(8) |
| Si8-O4 | 1.608(9) |
| Si8-O13 | 1.599(9) |
| Si8-O17 | 1.597(11) |
| Si8-O21 | 1.625(8) |
| Si9-O5 | 1.639(10) |
| Si9-O13 | 1.616(8) |
| Si9-O14 | 1.592(11) |
| Si9-O20 | 1.596(8) |
| Si10-O8 | 1.610(8) |
| Si10-O14 | 1.651(10) |
| Si10-O15 | 1.629(10) |
| Si10-O18 | 1.609(10) |
| Si11-O10 | 1.605(10) |
| Si11-O15 | 1.581(9) |
| Si11-O16 | 1.642(9) |
| Si11-O19 | 1.576(8) |
| Si-O (Avg.) | 1.597 |

**Table S5.** Bond angles for calcined, pure-silica PST-24 refined by the Rietveld method.

| Bond angle (°) | | | |
|---|---|---|---|
| O2-Si1-O2 | 103.3(7) | Si1-O2-Si4 (×2) | 174.6(5) |
| O2-Si1-O4 (×2) | 108.6(4) | Si1-O4-Si8 | 139.0(4) |
| O2-Si1-O6 (×2) | 111.9(4) | Si1-O6-Si7 | 140.3(4) |
| O4-Si1-O6 | 112.1(7) | Si2-O9-Si3 | 173.6(5) |
| O1-Si2-O1 | 100.4(6) | Si2-O1-Si5 (×2) | 162.4(8) |
| O1-Si2-O8 (×2) | 111.5(5) | Si2-O8-Si10 | 136.5(4) |
| O1-Si2-O9 (×2) | 110.3(5) | Si3-O9-Si2 | 173.6(5) |
| O8-Si2-O9 | 112.3(6) | Si3-O7-Si4 | 173.2(9) |
| O3-Si3-O3 | 115.5(6) | Si3-O3-Si6 (×2) | 168.2(6) |
| O3-Si3-O7 (×2) | 105.7(5) | Si4-O2-Si1 (×2) | 174.6(5) |
| O3-Si3-O9 (×2) | 112.9(5) | Si4-O7-Si3 | 173.2(9) |
| O7-Si3-O9 | 102.7(6) | Si4-O5-Si9 | 139.6(3) |
| O2-Si4-O2 | 112.0(7) | Si5-O1-Si2 (×2) | 162.4(8) |
| O2-Si4-O5 (×2) | 112.2(4) | Si5-O12-Si5 | 180 |
| O2-Si4-O7 (×2) | 106.9(4) | Si5-O11-Si6 | 147.7(7) |
| O5-Si4-O7 | 106.1(7) | Si6-O3-Si3 (×2) | 168.2(6) |
| O1-Si5-O1 | 109.0(5) | Si6-O11-Si11 | 147.7(7) |
| O1-Si5-O11 (×2) | 106.6(5) | Si6-O10-Si11 | 138.4(3) |
| O1-Si5-O12 (×2) | 109.4(5) | Si7-O6-Si1 | 140.3(4) |
| O11-Si5-O12 | 115.8(5) | Si7-O22-Si7 | 153.2(10) |
| O3-Si6-O3 | 100.7(5) | Si7-O17-Si8 | 149.2(7) |
| O3-Si6-O10 (×2) | 111.9(5) | Si7-O16-Si11 | 151.1(7) |
| O3-Si6-O11 (×2) | 110.0(5) | Si8-O4-Si1 | 139.0(4) |
| O10-Si6-O11 | 111.7(5) | Si8-O17-Si7 | 149.2(7) |
| O6-Si7-O16 | 107.9(7) | Si8-O21-Si8 | 155.3(11) |
| O6-Si7-O17 | 107.5(6) | Si8-O13-Si9 | 152.8(7) |
| O6-Si7-O22 | 115.3(7) | Si9-O5-Si4 | 139.6(3) |
| O16-Si7-O17 | 100.4(5) | Si9-O13-Si8 | 152.8(7) |
| O16-Si7-O22 | 112.2(8) | Si9-O20-Si9 | 153.4(13) |
| O17-Si7-O22 | 112.3(9) | Si9-O14-Si10 | 142.4(8) |
| O4-Si8-O13 | 109.4(7) | Si10-O8-Si2 | 136.5(4) |
| O4-Si8-O17 | 108.1(6) | Si10-O14-Si9 | 142.4(8) |
| O4-Si8-O21 | 114.1(7) | Si10-O18-Si10 | 145.7(16) |
| O13-Si8-O17 | 103.2(5) | Si10-O15-Si11 | 148.4(7) |
| O13-Si8-O21 | 109.5(7) | Si11-O10-Si6 | 138.4(3) |
| O17-Si8-O21 | 112.0(9) | Si11-O16-Si7 | 151.1(7) |
| O5-Si9-O13 | 106.2(6) | Si11-O15-Si10 | 148.4(7) |
| O5-Si9-O14 | 106.7(6) | Si11-O19-Si11 | 154.6(14) |
| O5-Si9-O20 | 113.8(8) | Si-O-Si (Avg.) | 153.1 |
| O13-Si9-O14 | 102.8(6) | | |
| O13-Si9-O20 | 111.5(7) | | |
| O14-Si9-O20 | 114.9(9) | | |
| O8-Si10-O14 | 106.7(6) | | |
| O8-Si10-O15 | 106.3(6) | | |

| | |
|---|---|
| O8-Si10-O18 | 112.1(8) |
| O14-Si10-O15 | 99.3(5) |
| O14-Si10-O18 | 121.6(12) |
| O15-Si10-O18 | 109.2(9) |
| O10-Si11-O15 | 108.1(5) |
| O10-Si11-O16 | 106.5(5) |
| O10-Si11-O19 | 116.1(8) |
| O15-Si11-O16 | 102.4(6) |
| O15-Si11-O19 | 112.2(8) |
| O16-Si11-O19 | 110.5(7) |
| O-Si-O (Avg.) | 109.3 |

**Table S6.** Fractional coordinates for polytypes PST-24A, PST-24B, and PST-24C.

| PST-24A ($P2_1/c$, $a$ = 25.3037 Å, $b$ = 10.1696 Å, $c$ = 23.7125 Å, $\beta$ = 128.6057°) | | | | | | | |
|---|---|---|---|---|---|---|---|
| Atom | *x* | *y* | *z* | Atom | *x* | *y* | *z* |
| Si1 | -0.3031 | 0.0262 | 0.2107 | O12 | -0.7734 | -0.0755 | -0.2798 |
| Si2 | -0.2859 | 0.0262 | 0.0927 | O13 | -0.7946 | -0.0629 | -0.1404 |
| Si3 | -0.8714 | -0.0269 | -0.1715 | O14 | -0.9133 | -0.0708 | -0.2545 |
| Si4 | -0.9524 | -0.0312 | -0.337 | O15 | -0.5484 | -0.0036 | -0.1979 |
| Si5 | -0.8405 | -0.0278 | -0.3556 | O16 | -0.8678 | 0.2467 | -0.5245 |
| Si6 | -0.8714 | 0.2231 | 0.3285 | O17 | -0.6699 | 0.0048 | 0.5752 |
| Si7 | -0.9524 | 0.2189 | 0.163 | O18 | -0.8991 | 0.1841 | 0.15 |
| Si8 | -0.8405 | 0.2223 | 0.1444 | O19 | -0.7734 | 0.1745 | 0.2202 |
| Si9 | -0.6969 | 0.2762 | -0.2107 | O20 | -0.6981 | 0.3165 | -0.145 |
| Si10 | -0.7141 | 0.2762 | -0.0927 | O21 | -0.7946 | 0.1872 | 0.3596 |
| Si11 | -0.0972 | -0.125 | 0.5771 | O22 | -0.9133 | 0.1793 | 0.2455 |
| Si12 | -0.0972 | 0.125 | 1.0771 | O23 | -0.0174 | -0.125 | 0.6137 |
| Si13 | -0.3996 | 0.125 | 0.2316 | O24 | -0.149 | -0.125 | 0.917 |
| Si14 | -0.3188 | 0.125 | 0.3985 | O25 | -0.1085 | -0.125 | 0.6349 |
| Si15 | -0.1604 | 0.125 | 0.4771 | O26 | -0.326 | -0.125 | 0.2014 |
| Si16 | -0.3996 | -0.125 | 0.7316 | O27 | -0.2976 | -0.125 | 0.0741 |
| Si17 | -0.3188 | -0.125 | 0.8985 | O28 | -0.0174 | 0.125 | 1.1137 |
| Si18 | -0.1604 | -0.125 | 0.9771 | O29 | -0.149 | 0.125 | 0.417 |
| Si19 | -0.5073 | -0.125 | 0.3101 | O30 | -0.1085 | 0.125 | 1.1349 |
| Si20 | -0.3568 | -0.125 | 0.4434 | O31 | -0.2407 | 0.125 | 0.4332 |
| Si21 | -0.5073 | 0.125 | -0.1899 | O32 | -0.3556 | 0.125 | 0.2058 |
| Si22 | -0.3568 | 0.125 | -0.0566 | O33 | -0.3552 | 0.125 | 0.3161 |
| O1 | -0.4516 | 0.2464 | 0.1979 | O34 | -0.2407 | -0.125 | 0.9332 |
| O2 | -0.1322 | -0.0034 | 0.5245 | O35 | -0.3556 | -0.125 | 0.7058 |
| O3 | -0.3301 | 0.2548 | 0.4248 | O36 | -0.3552 | -0.125 | 0.8161 |
| O4 | -0.3019 | 0.0665 | 0.145 | O37 | -0.1577 | -0.125 | 0.3722 |
| O5 | -0.4516 | -0.0036 | 0.6979 | O38 | -0.0302 | -0.125 | 0.3508 |
| O6 | -0.1322 | 0.2467 | 1.0245 | O39 | -0.1199 | -0.125 | 0.1636 |
| O7 | -0.3301 | 0.0048 | -0.0752 | O40 | -0.3258 | -0.125 | 0.5252 |
| O8 | -0.5484 | 0.2464 | 0.3021 | O41 | -0.4387 | -0.125 | 0.3903 |
| O9 | -0.8678 | -0.0034 | -0.0245 | O42 | -0.3258 | 0.125 | 0.0252 |
| O10 | -0.6699 | 0.2548 | 0.0752 | O43 | -0.4387 | 0.125 | -0.1097 |
| O11 | -0.8991 | -0.0659 | -0.35 | O44 | -0.5 | -0.125 | 0.25 |

| PST-24B ($P$-1, $a$ = 23.7125 Å, $b$ = 10.1696 Å, $c$ = 21.3024 Å, $\beta$ = 111.8378°) | | | | | | | |
|---|---|---|---|---|---|---|---|
| Atom | *x* | *y* | *z* | Atom | *x* | *y* | *z* |
| Si1 | 0.5501 | 0.0981 | 0.1286 | O24 | 0.0933 | 0.1284 | 0.8678 |
| Si2 | 0.6346 | 0.0939 | 0.0476 | O25 | 0.9951 | 0.3702 | 0.6699 |
| Si3 | 0.7651 | 0.0973 | 0.1595 | O26 | 0.7991 | 0.1909 | 0.8991 |
| Si4 | 0.2638 | 0.1512 | 0.3031 | O27 | 0.7436 | 0.2005 | 0.7734 |
| Si5 | 0.1286 | 0.1512 | 0.2859 | O28 | 0.9042 | 0.1879 | 0.7946 |
| Si6 | 0.7638 | 0.4012 | 0.3031 | O29 | 0.9088 | 0.1958 | 0.9133 |
| Si7 | 0.6286 | 0.4012 | 0.2859 | O30 | 0.3995 | 0.1286 | 0.4516 |
| Si8 | 0.7362 | 0.1512 | 0.6969 | O31 | 0.4067 | 0.3784 | 0.1322 |
| Si9 | 0.8714 | 0.1512 | 0.7141 | O32 | 0.5049 | 0.1202 | 0.3301 |
| Si10 | 0.9499 | 0.1519 | 0.8714 | O33 | 0.7009 | 0.4409 | 0.1009 |
| Si11 | 0.8654 | 0.1562 | 0.9524 | O34 | 0.7564 | 0.4505 | 0.2266 |
| Si12 | 0.7349 | 0.1528 | 0.8405 | O35 | 0.5958 | 0.4379 | 0.2054 |
| Si13 | 0.5501 | 0.4019 | 0.1286 | O36 | 0.5912 | 0.4458 | 0.0867 |
| Si14 | 0.6346 | 0.4062 | 0.0476 | O37 | 0.8995 | 0.3786 | 0.4516 |
| Si15 | 0.7651 | 0.4028 | 0.1595 | O38 | 0.9067 | 0.1284 | 0.1322 |
| Si16 | 0.0501 | 0.1519 | 0.1286 | O39 | 0.0049 | 0.3702 | 0.3301 |
| Si17 | 0.1346 | 0.1562 | 0.0476 | O40 | 0.2009 | 0.1909 | 0.1009 |
| Si18 | 0.2651 | 0.1528 | 0.1595 | O41 | 0.2564 | 0.2005 | 0.2266 |
| Si19 | 0.7638 | 0.0988 | 0.3031 | O42 | 0.6969 | 0.0585 | 0.3019 |
| Si20 | 0.6286 | 0.0989 | 0.2859 | O43 | 0.0958 | 0.1879 | 0.2054 |
| Si21 | 0.4243 | 0 | 0.0972 | O44 | 0.0912 | 0.1958 | 0.0867 |
| Si22 | 0.9243 | 0.25 | 0.0972 | O45 | 0.3811 | 0 | 0.0174 |
| Si23 | 0.0757 | 0.25 | 0.9028 | O46 | 0.816 | 0 | 0.149 |
| Si24 | 0.3812 | 0.25 | 0.3996 | O47 | 0.4934 | 0 | 0.1085 |
| Si25 | 0.4673 | 0.25 | 0.3188 | O48 | 0.2774 | 0 | 0.326 |
| Si26 | 0.3875 | 0.25 | 0.1604 | O49 | 0.1217 | 0 | 0.2976 |
| Si27 | 0.8812 | 0 | 0.3996 | O50 | 0.8811 | 0.25 | 0.0174 |
| Si28 | 0.9673 | 0 | 0.3188 | O51 | 0.316 | 0.25 | 0.149 |
| Si29 | 0.8875 | 0 | 0.1604 | O52 | 0.9934 | 0.25 | 0.1085 |
| Si30 | 0.6188 | 0.25 | 0.6004 | O53 | 0.7774 | 0.25 | 0.326 |
| Si31 | 0.5327 | 0.25 | 0.6812 | O54 | 0.6217 | 0.25 | 0.2976 |
| Si32 | 0.6125 | 0.25 | 0.8396 | O55 | 0.1189 | 0.25 | 0.9826 |
| Si33 | 0.5674 | 0 | 0.5073 | O56 | 0.684 | 0.25 | 0.851 |
| Si34 | 0.5502 | 0 | 0.3568 | O57 | 0.0066 | 0.25 | 0.8915 |
| Si35 | 0.0674 | 0.25 | 0.5073 | O58 | 0.4239 | 0.25 | 0.2407 |
| Si36 | 0.0502 | 0.25 | 0.3568 | O59 | 0.3114 | 0.25 | 0.3556 |
| Si37 | 0.9326 | 0.25 | 0.4927 | O60 | 0.4213 | 0.25 | 0.3552 |
| Si38 | 0.9498 | 0.25 | 0.6432 | O61 | 0.7799 | 0.25 | 0.1577 |
| Si39 | 0.4243 | 0.5 | 0.0972 | O62 | 0.631 | 0.25 | 0.0302 |
| Si40 | 0.8812 | 0.5 | 0.3996 | O63 | 0.5335 | 0.25 | 0.1199 |
| Si41 | 0.9673 | 0.5 | 0.3188 | O64 | 0.9239 | 0 | 0.2407 |
| Si42 | 0.8875 | 0.5 | 0.1604 | O65 | 0.8114 | 0 | 0.3556 |
| Si43 | 0.5674 | 0.5 | 0.5073 | O66 | 0.9213 | 0 | 0.3552 |
| Si44 | 0.5502 | 0.5 | 0.3568 | O67 | 0.2799 | 0 | 0.1577 |
| O1 | 0.3995 | 0.3714 | 0.4516 | O68 | 0.131 | 0 | 0.0302 |
| O2 | 0.4067 | 0.1217 | 0.1322 | O69 | 0.0335 | 0 | 0.1199 |
| O3 | 0.5049 | 0.3798 | 0.3301 | O70 | 0.5761 | 0.25 | 0.7593 |
| O4 | 0.7009 | 0.0591 | 0.1009 | O71 | 0.6886 | 0.25 | 0.6444 |
| O5 | 0.7564 | 0.0495 | 0.2266 | O72 | 0.5787 | 0.25 | 0.6448 |
| O6 | 0.1969 | 0.1915 | 0.3019 | O73 | 0.601 | 0 | 0.3258 |
| O7 | 0.5958 | 0.0622 | 0.2054 | O74 | 0.579 | 0 | 0.4387 |
| O8 | 0.5912 | 0.0543 | 0.0867 | O75 | 0.101 | 0.25 | 0.3258 |
| O9 | 0.8995 | 0.1214 | 0.4516 | O76 | 0.079 | 0.25 | 0.4387 |

| | | | | | | | |
|---|---|---|---|---|---|---|---|
| O10 | 0.9067 | 0.3717 | 0.1322 | O77 | 0.899 | 0.25 | 0.6742 |
| O11 | 0.0049 | 0.1298 | 0.3301 | O78 | 0.921 | 0.25 | 0.5613 |
| O12 | 0.6969 | 0.4415 | 0.3019 | O79 | 0 | 0.25 | 0.5 |
| O13 | 0.6005 | 0.3714 | 0.5484 | O80 | 0.3811 | 0.5 | 0.0174 |
| O14 | 0.5933 | 0.1217 | 0.8678 | O81 | 0.816 | 0.5 | 0.149 |
| O15 | 0.4951 | 0.3798 | 0.6699 | O82 | 0.4934 | 0.5 | 0.1085 |
| O16 | 0.8031 | 0.1915 | 0.6981 | O83 | 0.9239 | 0.5 | 0.2407 |
| O17 | 0.1005 | 0.1214 | 0.5484 | O84 | 0.8114 | 0.5 | 0.3556 |
| O18 | 0.0933 | 0.3717 | 0.8678 | O85 | 0.9213 | 0.5 | 0.3552 |
| O19 | 0.9951 | 0.1298 | 0.6699 | O86 | 0.601 | 0.5 | 0.3258 |
| O20 | 0.6005 | 0.1286 | 0.5484 | O87 | 0.579 | 0.5 | 0.4387 |
| O21 | 0.5933 | 0.3784 | 0.8678 | O88 | 0.5 | 0 | 0.5 |
| O22 | 0.4951 | 0.1202 | 0.6699 | O89 | 0.5 | 0.5 | 0.5 |
| O23 | 0.1005 | 0.3786 | 0.5484 | | | | |

| PST-24C ($P2/c$, $a$ = 25.3037 Å, $b$ = 10.1696 Å, $c$ = 23.7125 Å, $\beta$ = 128.6057°) | | | | | | | |
|---|---|---|---|---|---|---|---|
| Atom | *x* | *y* | *z* | Atom | *x* | *y* | *z* |
| Si1 | -0.1286 | 0.2231 | 0.1715 | O13 | -0.5484 | 0.2536 | -0.1979 |
| Si2 | -0.0476 | 0.2189 | 0.337 | O14 | -0.6699 | 0.2452 | 0.5752 |
| Si3 | -0.1595 | 0.2223 | 0.3556 | O15 | -0.0174 | -0.125 | 0.6137 |
| Si4 | -0.6969 | 0.2238 | 0.2893 | O16 | -0.149 | -0.125 | 0.917 |
| Si5 | -0.7141 | 0.2239 | 0.4073 | O17 | -0.1085 | -0.125 | 0.6349 |
| Si6 | -0.0972 | -0.125 | 0.5771 | O18 | -0.2407 | 0.125 | 0.4332 |
| Si7 | -0.3996 | 0.125 | 0.2316 | O19 | -0.3556 | 0.125 | 0.2058 |
| Si8 | -0.3188 | 0.125 | 0.3985 | O20 | -0.3552 | 0.125 | 0.3161 |
| Si9 | -0.1604 | 0.125 | 0.4771 | O21 | -0.3258 | -0.125 | 0.5252 |
| Si10 | -0.5073 | -0.125 | 0.3101 | O22 | -0.4387 | -0.125 | 0.3903 |
| Si11 | -0.3568 | -0.125 | 0.4434 | O23 | -0.1322 | 0.4967 | 0.5245 |
| Si12 | -0.1286 | 0.4731 | 0.6715 | O24 | -0.1009 | 0.4341 | 0.85 |
| Si13 | -0.0476 | 0.4689 | 0.837 | O25 | -0.2266 | 0.4245 | 0.7798 |
| Si14 | -0.1595 | 0.4723 | 0.8556 | O26 | -0.3019 | 0.5665 | 0.145 |
| Si15 | -0.3031 | 0.5262 | 0.2107 | O27 | -0.2054 | 0.4372 | 0.6404 |
| Si16 | -0.2859 | 0.5262 | 0.0927 | O28 | -0.0867 | 0.4293 | 0.7545 |
| Si17 | -0.0972 | 0.375 | 0.5771 | O29 | -0.4516 | 0.4964 | 0.6979 |
| Si18 | -0.3996 | 0.625 | 0.2316 | O30 | -0.3301 | 0.5048 | -0.0752 |
| Si19 | -0.3188 | 0.625 | 0.3985 | O31 | -0.0174 | 0.375 | 0.6137 |
| Si20 | -0.1604 | 0.625 | 0.4771 | O32 | -0.149 | 0.375 | 0.917 |
| Si21 | -0.5073 | 0.375 | 0.3101 | O33 | -0.1085 | 0.375 | 0.6349 |
| Si22 | -0.3568 | 0.375 | 0.4434 | O34 | -0.326 | 0.375 | 0.2014 |
| O1 | -0.4516 | 0.2464 | 0.1979 | O35 | -0.2976 | 0.375 | 0.0741 |
| O2 | -0.1322 | -0.0034 | 0.5245 | O36 | -0.2407 | 0.625 | 0.4332 |
| O3 | -0.3301 | 0.2548 | 0.4248 | O37 | -0.3556 | 0.625 | 0.2058 |
| O4 | -0.4516 | -0.0036 | 0.6979 | O38 | -0.3552 | 0.625 | 0.3161 |
| O5 | -0.1322 | 0.2467 | 1.0245 | O39 | -0.1577 | 0.625 | 0.8722 |
| O6 | -0.3301 | 0.0048 | -0.0752 | O40 | -0.0302 | 0.625 | 0.8508 |
| O7 | -0.1009 | 0.1841 | 0.35 | O41 | -0.1199 | 0.625 | 0.6636 |
| O8 | -0.2266 | 0.1745 | 0.2798 | O42 | -0.3258 | 0.375 | 0.5252 |
| O9 | -0.2054 | 0.1872 | 0.1404 | O43 | -0.4387 | 0.375 | 0.3903 |
| O10 | -0.0867 | 0.1793 | 0.2545 | O44 | -0.5 | -0.125 | 0.25 |
| O11 | -0.8678 | 0.2534 | -0.0245 | O45 | -0.5 | 0.375 | 0.25 |
| O12 | -0.6981 | 0.1835 | 0.355 | | | | |

## References


[S1] J. E. Schmidt, M. A. Deimund, D. Xie, M. E. Davis, *Chem. Mater*. **2015**, *27*, 3756-3762.

[S2] A. Rojas, O. Arteaga, B. Kahr, M. A. Camblor, *J. Am. Chem. Soc.* **2013**, *135*, 11975-11984.

[S3] W. Wan, J. Sun, J. Su, S. Hovmöller, X. Zou, *J. Appl. Cryst.* **2013**, *46*, 1863-1873.

[S4] D. Zhang; P. Oleynikov, S. Hovmöller; X. Zou. *Z. Kristallogr.* **2010**, *225,* 94-102.

[S5] W, Kabsch, *Acta Cryst.* **2010**, *D66*, 125-132.

[S6] G. M. Sheldrick, *Acta Cryst.* **2015**, *A71*, 3-8.

[S7] E. J. Kirkland, B. M. Siegel, *Ultramicroscopy* **1980**, *5*, 479-503.

[S8] W. Wan, S. Hovmöller, X. Zou, *Ultramicroscopy* **2012**, *115,* 50-60.

[S9] H. M. Rietveld, *J. Appl. Crystallogr.* **1969**, *2*, 65-71.

[S10] A. C. Larson, R. B. Von Dreele, *General Structure Analysis System (GSAS), Los Alamos National Laboratory Report LAUR 86-748*, Los Alamos National Laboratory: Los Alamos, NM, 2004.

[S11] B. H. Toby, *J. Appl. Crystallogr.* **2001**, *34,* 210-213.

[S12] W. H. Baur, R. X. Fischer, *Chem. Mater.* **2019**, *31*, 2401-2420.

[S13] J. B. Hastings, W. Thomlinson, D. E. Cox, *J. Appl. Crystallogr.* **1984**, *17*, 85-95.

[S14] Dassault Systémes BIOVIA, Materials Studio, San Diego: Dassault Systémes, 2017.

[S15] M. D. Foster, A. Simperler, R. G. Bell, O. D. Friedrichs. F. A. A. Paz, J. Klinowski, *Nat. Mater.* **2004**, *3*, 234-238.

[S16] K.-P. Schröder, J. Sauer, M. Leslie, C. R. A. Catlow, J. M. Thomas, *Chem. Phys. Lett.* **1992**, *188*, 320-325.

[S17] J. D. Gale, A. L. Rohl, *Mol. Simul.* **2003**, *29*, 291-341.

[S18] W. Wan, CrystDiff, https://www.mmk.su.se/zou/electron-crystallography-software/crystdiff.

[S19] S. L. Mayo, B. D. Olafson, W. A. Goddard III, *J. Phys. Chem.* **1990**, *94*, 8897-8909.
